# Study on the Extrapolability of Current Tumorgenicity Test With Mice by Comparing the Syngeneic or Allogeneic Mouse Transplantation Model

**DOI:** 10.1093/stcltm/szae019

**Published:** 2024-06-10

**Authors:** Takashi Tamura, Tsuyoshi Tahara, Michiko Inoue, Ryota Nanjo, Hirotaka Onoe, Takako Yamamoto, Shin Kawamata

**Affiliations:** Department of Science, Technology and Innovation, Kobe University, Japan; Division of Bio-function Dynamics Imaging, RIKEN Center for Life Science Technology, and RIKEN Center for Biosystems Dynamics Research, Japan; Department of In Vivo Imaging, Advanced Research Promoting Center, Tokushima University, Japan; Division of Bio-function Dynamics Imaging, RIKEN Center for Life Science Technology, and RIKEN Center for Biosystems Dynamics Research, Japan; Department of Science, Technology and Innovation, Kobe University, Japan; Division of Bio-function Dynamics Imaging, RIKEN Center for Life Science Technology, and RIKEN Center for Biosystems Dynamics Research, Japan; Human Brain Research Center, Graduate School of Medicine, Kyoto University, Japan; Department of Science, Technology and Innovation, Kobe University, Japan; Department of Science, Technology and Innovation, Kobe University, Japan; Cyto-Facto Inc., Japan

**Keywords:** extrapolability, tumorigenicity test, syngeneic transplantation, allogeneic transplantation, NOG mouse, induced pluripotent stem cell

## Abstract

The extrapolability of the current tumorigenicity test performed by transplanting human cell product into immunodeficient (NOG) mice was investigated. For this purpose, the susceptibility to form teratomas of NOG mice was assessed by transplanting undifferentiated human-induced pluripotent stem cells (hiPSCs) as positive control cells via the liver, striatum, or tail vein and evaluating the TPD50 value (dose required to form teratomas in half of the transplanted mice). This was then compared to the TPD50 of syngeneic or allogeneic mouse models. The TPD50 of C57/BL/6(B6)-iPSC or 129/Ola(129)-embryonic stem cell (ESC) transplanted into the liver of syngeneic mice was 4.08 × 10^5^ and 4.64 × 10^4^ cells, respectively, while the TPD50 of hiPSC administered into the liver of NOG mice was 4.64 × 10^4^ cells. The TPD50 of B6-miPSC-synergic, 129-mESC-synergic, or 129-cell/B6 allogeneic transplantation into the striatum was 5.09 × 10^2^, 1.0 × 10^4^, and 3.73 × 10^4^ cells, respectively, while that of hiPSC/NOG mice was 1.0 × 10^3^ cells. The TPD50 for B6-miPSC or 129-mESC syngeneic tail vein infusion was 3.16 × 10^6^ or 5.62 × 10^6^ cells, respectively, while no incidence was observed from 1 × 10^7^ B6-miPSCs in 129 mice or hiPSCs in NOG mice infusion study. Although the number of data sets was limited, these data indicate that the teratoma formation from transplanted undifferentiated hiPSCs via the liver or striatum in NOG mice is comparable to that in syngeneic or allogeneic mouse transplantation model, suggesting that the result of the current tumorigenicity test in NOG mice would provide useful information to infer the incidence of teratoma from residual undifferentiated hPSCs in hPSC-derived products after transplantation.

## Graphical Abstract

**Graphical Abstract:**
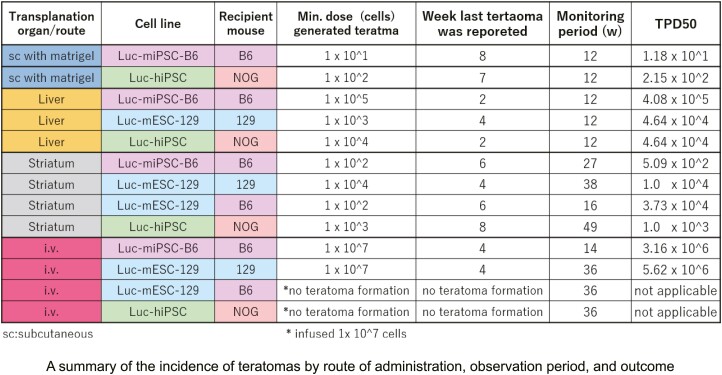

**Significance Statement**

The extrapolability of the tumorigenicity test of human-induced pluripotent stem cell (hiPSC)-derived cells was investigated. The susceptibility of transplanted undifferentiated hiPSCs to generate teratomas in the liver, striatum, or tail vein of NOG mice was assessed by TPD50 values (the dose required to generate teratomas in half of the transplanted mice). The TPD50 values of relevant syngeneic mouse transplantation models were then compared. Our data indicated that the frequency of teratoma formation in NOG mice is comparable to that in the syngeneic mouse transplantation model, suggesting that the current tumorigenicity assay is still useful to provide information on the safety of hiPSC-based cell therapy.

## Introduction

Pluripotent stem cell (PSC)-based cell therapies, such as Parkinson’s disease for induced pluripotent stem cell (iPSC)-derived dopamine precursor,^1,2^ chronic heart failure for iPSC-derived cardiomyocytes,^3^ age-related macular degeneration for iPSC-derived retinal pigment epithelium,^4,5^ anemia for iPSC-derived red blood cells,^6,7^ thrombocytopenia for iPSC-derived platelets,^8,9^ solid tumor for iPSC-derived chimeric antigen receptor T cells,^10^ iPSC-derived natural killer cells,^11^ congenital liver disease for embryonic stem cell-derived hepatocytes^12,13^ have been reported elsewhere.

Meanwhile, PSC-derived final products sometimes contain differentiation-resistant undifferentiated cells or not fully differentiated progenitor-like cells due to genetic abnormalities or epigenetic dysregulation acquired during prolonged culture periods or incomplete reprogramming in the case of iPSC-derived products. These cells have the potential to generate off-target tissues including tumors or teratomas after transplantation.^14,15^ However, we do not have sufficient knowledge to correlate a genetic or epigenetic abnormality with a corresponding tumorigenic event. In addition, we must consider the contribution of the microenvironment into which the cells are transplanted to determine cell fate after transplantation.^16-18^ These issues indicate that the phenotype or behavior of cells after transplantation is unpredictable with in vitro assays alone and need to conduct tumorigenicity test via clinical route using immunodeficient rodents to predict the incidence of abnormal tissue formation after transplantation of PSC-derived cell product especially in the case of first-in-human (FIH) trial. Thus, in vivo animal studies can coexist with and complement in vitro assays, and several tumorigenicity tests for respective iPSC-derived final products have been reported.^16-20^ However, in the absence of standardized in vivo assay systems and available interpretation, a case-by-case approach is required to address this issue.^21^

Then, we come to the long-standing question of how reliable the result of a tumorigenicity test using immunodeficient mice (xenograft model) is for predicting a clinical outcome. Namely, the “extrapolarity” of the current tumorigenicity test which involves transplanting human iPSC-derived cells into immunodeficient mice needs to be assessed. While the importance of addressing the “extrapolability” or validity of the current tumorigenicity test for predicting the clinical outcome of hPSC-derived cell therapy is well recognized, there are few studies or reports available to date. In addition, the sensitivity for detecting transplanted cells in deep organs in mice needs to be improved for a fair evaluation of the tumorigenic event, as the sensitivity of the widely accepted Alu-PCR method for detecting human cells in mouse organs was as low as 0.1-0.001%.^19,22^ The sensitivity for the detection of human cells using Alu-PCR varies with different analytical methods, and the Alu-PCR result varies greatly depending on the location of the dissected tissue block, which may not provide reliable information on the biodistribution of transplanted human cells in mouse organs.

Therefore, a study of the extrapolability of current tumorigenicity assays was initiated by exploring new methods to detect transplanted human cells in mouse organs.

### Experimental Procedures

All experiments involving human (hiPSC) and mouse ESC (mESC) samples in this study were reviewed and approved by the Ethics Committee of the Foundation for Biomedical Research and Innovation in Kobe (FBRI) (No. 18-05-02) and Cyto-Facto Inc. (No.23-5-1). This study does not involve human subjects or the establishment of a new iPSC cell line from human tissue. All experimental protocols involving mice were approved by the Animal Experimentation Committee of RIKEN BDR (MA2006-09-19).

#### Generation of Luciferase Gene Containing Contract pCAG-FlucIP

The entry vector, pCAGIPuro-GRFC, was a gift from Dr. Y. Sasai (RIKEN Centre for Developmental Biology, Japan). The firefly luciferase2 (luc2) gene fragment containing the attB sequence at both ends was amplified by PCR from the pGL4.17[luc2/Neo] vector (accession number: DQ188837, Promega) and then cloned into the Gateway donor vector pDonr221 by the BP Clonase reaction to generate pDonr-luc2 (Invitrogen). The firefly luciferase gene expression vector pCAG-FlucIP was generated by a recombination reaction between pDonr-Luc and pCAGIPuro-GRFC using LR Clonase (Invitrogen).

#### Cell Culture for Stable Luciferase-Transfected Cell Lines

C57BL/6 (B6) mouse-derived mouse iPSC (miPSC) line 956-C1-1 (kindly provided by Dr K. Okita, Kyoto Uni. CiRA) cells, designated as miPSC-B6, were maintained in a medium consisting of DMEM (Gibco), 15% FBS (JRH), 2 mM l-glutamine (Nacalai), NEAA (Gibco, 1:100 dilution), 10 units/10 μg/mL penicillin/streptomycin (Gibco), StemSure mLIF (Fujifilm, 1:1000 dilution), and 55 μM 2-mercaptoethanol (Gibco). Mouse 129/Ola-derived mESC line EB5^23,24^ (purchased from RIKEN BRC) cells, hereafter referred to as mESC-129, was maintained in GMEM (Gibco), 10% KSR (Gibco), 1% FBS (JRH), 2 mM l-glutamine (Nacalai), NEAA (Gibco, 1:100 dilution), 10 units/10 μg/mL penicillin/streptomycin (Gibco), StemSure mLIF (Fujifilm, 1:1000 dilution), 55 μM 2-mercaptoethanol (Gibco), and 20 μg/mL blastcidin (Wako, 1:500 dilution). The medium was changed daily. Cells were passaged by seeding a single-cell suspension with 0.25% trypsin-EDTA (Gibco). Cells were seeded at 2 × 10^6^ cells in a 100-mm dish coated with 0.1% gelatin (Sigma) and incubated at 37 °C in an incubator with 5% CO_2_ and 95% humidity.

Human iPSC line PFX#9,^25^ hereafter referred to as human iPSC (hiPSC)-PFX#9, was cultured in the primed state with chemical defined medium Essential 8 (Thermo Fisher Scientific) on recombinant human Vitronectin-N (Thermo Fisher Scientific)-coated dishes. Cells were cultured for 3 days and passaged by seeding in a single-cell suspension using TrypLE Select (Thermo Fisher Scientific). Cells were seeded at 1 × 10^5^ cells/well in the presence of ROCK inhibitor Y-27632 (10 μM) in 6-well plates in the presence of the ROCK inhibitor and cultured for 1 day, followed by culture with Essentia 8 without ROCK inhibitor with daily medium changes. Cells were incubated at 37 °C in an atmosphere containing 5% CO_2_.

Amaxa 4D-Nucleofector (Lonza) was used to generate stable FlucIP transfectant, according to the manufacturer’s protocol. Briefly, miPS-B6 and mESC-129 cells from were harvested using trypsin, and 5 × 10^6^ cells were resuspended in 100 mL of nucleofection buffer. Then, 20 mg of pCAG-FlucIP plasmid digested with *Pvu*I (New England Biolabs, #R3150) for linearization was added to the nucleofection buffer. The resulting nucleofection buffer-cell-DNA mixture was nucleofected with a 4D-Nucleofector using program CG-104 according to the manufacturer’s recommendations. Cells were harvested and cultured in 0.1% gelatin (Sigma) coated 100 mm dish. For hiPSC-PFX#9, 8 × 10^6^ cells and 5 mg *Pvu*I linearized pCAG-FlucIP plasmids were mixed and then nucleofected using CB-150 program. After 2 days of incubation, cells containing firefly luciferase gene fragment were selected in the presence of 1 mg/mL puromycin (InvivoGen) for 48 hours to establish FlucIP gene containing transfectants Luc-miPSC-B6, Luc-mESC-129, or Luc-hiPSC-PFX#9, respectively. Puromycin selected colonies were collected and passaged for further expansion.

#### Animal Experiments

C57BL/6 mice at 4 weeks of age were purchased from CLEA Japan Inc. (Shizuoka, Japan), 129/Sv mice at 6-8 weeks of age were purchased from RIKEN BRC and NOG mice (NOD.Cg-PrkdcscidIl2rgtm1Sug/ShiJic)^26^ of 6 weeks of age were purchased from In Vivo Science Inc. (Kanagawa, Japan). Various doses of the designated cells were transplanted into mice by clinical routes. All mice were bred and monitored at the Riken Animal Facility until the end of the experiment.

#### Subcutaneous Transplantation

Luc-miPS-B6 cells (1 × 10^1-3^ cells/50 µL) were mixed with 200 µL growth factor-containing basement Matrigel matrix (Corning; 354234) and then injected subcutaneously into the back of B6 mice. Teratoma formation was monitored weekly for 12 weeks.

#### Liver Transplantation

Luc-miPSC-B6, Luc-mESC-129, or Luc-hiPS-PFX#9 cells (1 × 10^1-6^ cells/10 µL) were injected into the left lobe of mouse liver under deep anesthesia.

For the Luc-hiPS-PFX#9 injection, ROCK inhibitor Y-27632 (10uM) was added to the cell culture 60 minutes before injection and harvested in a single-cell suspension for injection. Sixty minutes after cell injection, 3 mg of D-luciferin in 200 mL of PBS (−) was administered intraperitoneally and then the fluorescence signal was captured with the IVIS in vivo imaging system (PerkinElmer) and analyzed with the Living Image 3.2 software (PerkinElmer) The longest observation period was 12 weeks.

#### Brain Striatum Transplantation

Luc-miPSC-B6, Luc-mESC-129 (1 × 10^1-5^ cells/4 µL), or Luc-hiPS-PFX#9 cells (1 × 10^2-6^ cells/4 µL) were directly injected into the left striatum of mice under deep anesthesia. For the Luc-hiPS-PFX#9 injection, ROCK inhibitor Y-27632 (10 µM) was added to the cell culture 60 minutes before injection and harvested into a single-cell suspension for injection. Sixty minutes after cell injection, 3 mg of d-luciferin in 200 mL of PBS (−) was administered intraperitoneally and then IVIS imaging was performed. The longest observation period was 27 weeks for Luc-miPSC-B6 transplanted B6 mice, 38 weeks for Luc-mESC-129 transplanted 129 mice, 16 weeks for Luc-mESC-129 transplanted B6 mice, and 49 weeks for Luc-hiPSC-PFX#9 transfected NOG mice.

#### Tail Vein Infusion

Luc-miPSC-B6, Luc-mESC-129, or Luc-hiPS-PFX#9 cells (1 × 10^5-7^ cells) in 200 µL PBS (−) were infused via mouse tail vein. Luc-hiPS-PFX#9 cells were suspended in a single cell in the presence of ROCK inhibitor (Y-27632) at the concentration of 10 mM 60 minutes before injection, washed again, and resuspended in 200 mL of PBS (−) for injection. Sixty minutes after cell injection, 3 mg of d-luciferin in 200 mL of PBS (−) was administered intraperitoneally and then IVIS imaging was performed shortly after infusion. The observation period for Luc-miPSC-B6 infused B6 mice was 14 weeks, for Luc-miPSC-B6 or Luc-mESC-129 infused 129 mice was 6 weeks and for Luc-hiPSC-PFX#9 infused NOG mice was 24 weeks.

#### Quantitative RT-PCR and droplet digital PCR

Total RNA from the mixture of human iPSC PFX#9 (FBRI) and hepatocytes (HUCPG, Lonza) or astrocytes (CC-2565, Lonza) or peripheral blood cells (PBMC) (70025.1, STEMCELL) at a ratio of 1:1 × 10^2^, 1 × 10^3^, 1 × 10^4^, 1 × 10^5^ was extracted using a Rneasy micro kit (74004, QIAGEN) according to the manufacturer’s instructions. 0.5 μg of total RNA was used for cDNA synthesis using the QuantiTect Reverse Transcription Kit (205311, Qiagen). Quantitative PCR (qPCR) was performed with TaqMan probes for GAPDH (for internal control; Hs02786624_g1, Thermo Fisher Scientific), POU5F1 (Hs04260367_gH, Thermo Fisher Scientific), LIN28A (Hs00702808_s1, Thermo Fisher Scientific), and PRDM14 (Hs01119056_m1, Thermo Fisher Scientific) using a qRT-PCR device (StepOnePlus, Life Technologies).

The detection limit for the copy number of LIN28A from iPSCs, hepatocytes, astrocytes, and PBMC was determined by digital droplet PCR (Bio-Rad Laboratories, Hercules, CA, USA). Briefly, cDNA was synthesized from 5 ng total RNA extracted from cells using TaqMan gene expression assays. The PCR mix containing the TaqMan probe for LIN28A (Hs00702808_s1) was loaded into a Bio-Rad QX-100 emulsification device, and droplets were formed according to the manufacturer’s instructions. After cycling, raw fluorescence data for each well were exported from the manufacturer’s software (Bio-Rad QuantaSoft v. 1.2) for analysis.

#### Statistics for TPD50 Calculation

TPD50 values were calculated at each time point using the Spearman-Kärber method.^27^

## Results

### Cell Mass of 1 × 10^3^ Cells Can Be Detected in Liver When Labeled With FlucIP

**Figure 1.**
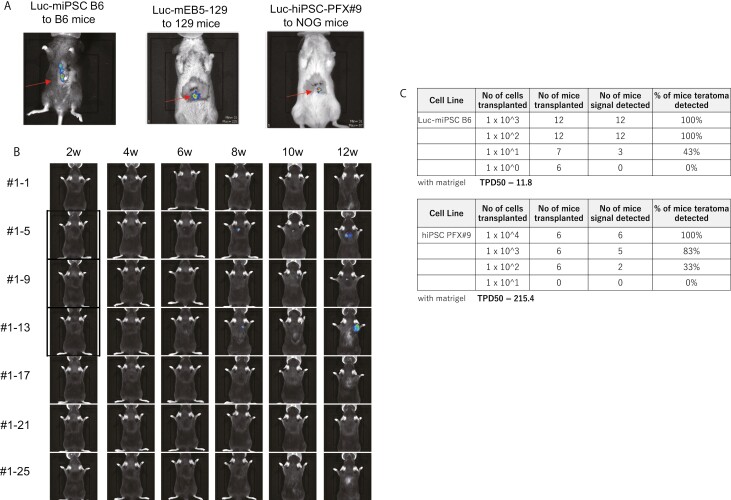
Minimum number of transplanted iPSCs detected in mice. (**A**) Signals from 1 × 10^3^ Luc-miPSC-B6 cells, mESC-129 cells, or hiPSC-PFX#9 transplanted into the liver of B6, 129, or NOG mice, respectively. Signals were acquired 60 minutes after cell administration. (**B**) Biweekly IVIS images of B6 mice subcutaneously transplanted with 1 × 10^1^ syngeneic Luc-labeled miPSC-B6 cells. Mice developed teratomas were marked with rectangles. The observation period was 2-12 weeks. (**C**) The result of tumorigenicity tests for miPSC-B6 cells (upper table) and hiPSC-PFX#9 (lower table), and calculated TPD50 values.

To increase the detection sensitivity of small cell masses transplanted into deep organs, the cells to be transplanted were genetically labeled with the luciferase construct FlucIP and the detection sensitivity of FlucIP-labeled cells (hereafter referred to as Luc cells) was determined. Multiple doses of Luc-miPSC-C57BL/6 (Luc-miPSC-B6) were transplanted into B6 mice (syngeneic transplantation model) or Luc-mESC-129/Ola (Luc-mESC-129) into 129 mice (syngeneic transplantation model), or Luc-hiPSC-PFX#9 into NOG mice (xenograft model), and 60 minutes after transplantation of the cells into the left lobe of the liver, luciferin was administered intraperitoneally and the signals from the transplanted cells were observed using the IVIS imaging system. The minimum cell dose to detect signal from labeled cells in the liver was 1 × 10^3^ cells or less for both the syngeneic and xenograft models (Fig. 1A). The detection of cell mass of 1 × 10^3^ cells or more in deep organs such as the liver is useful for making a fair decision regarding the presence of a cell mass in tumorigenicity test or biodistribution test taking into account the size of the cell mass and the size of the cell mouse liver when cells are genetically labeled with FlucIP.

### Study Design to Evaluate the Extrapolability of the Current Tumorigenicity Test

After evaluating the sensitivity of cell detection in deep organs using the FlucIP labeling system, we investigated the extrapolability of the current tumorigenicity test using NOG mice was investigated. To address this issue, we transplanted different doses of undifferentiated hiPSC into NOG mice via the liver, striatum, or tail vein infusion as positive control cells capable of producing detectable cell mass, in this case teratomas, and evaluated the susceptibility of NOG mice to produce detectable cell mass by calculating the TPD50 value (the minimum dose of cells to produce cell mass in half of the transplanted animals). The TPD50 values of human undifferentiated iPSCs with NOG mice were then compared to those of syngeneic or allogeneic mouse transplantation models by transplanting undifferentiated PSCs, assuming that the survival of ROCK inhibitor-treated human iPSCs and mouse PSCs in the transplanted mouse microenvironment is comparable and that the syngeneic or allogeneic mouse transplantation model would recapitulate the clinical outcome of autologous or allogeneic transplantation with hPSC-derived products. Based on these premises, we attempted to assess the extrapolability of the current tumorigenicity test by comparing the TPD50 value of this test with that of the syngeneic or allogeneic mouse transplantation model.

We selected mouse strains B6 (black-haired) and 129 (brown-haired) with the same major histocompatibility complex (MHC) background (H-2Kb, H-2Db, H-2Lnull, I-Ab, and I-Enull) and different minor antigens among 44 mouse strains to perform syngeneic transplantation that could recapitulate autologous transplantation and a minor antigen-mismatched “modulated” allogeneic transplantation that could recapitulate the moderate GvHD after BM transplantation by HLA identical with minor antigens different donor^28^ and a “controlled” allogeneic transplantation in the clinic using immunosuppressants. We included this “modulated” allogeneic transplantation in the experimental design with the expectation that this model would provide useful insight to evaluate the susceptibility of NOG mice to generate human teratomas compared to a mild rejection mouse model rather than a simple MHC-mismatched allogeneic rejection model, considering the clinical setting. In this experimental design, we expected that the TPD50 value of the syngeneic mPSC transplantation model would be lower than that of the “modulated” allogeneic or xeno transplantation model using immunodeficient NOG mice, but we did not expect that the TPD50 of the “modulated” allogeneic transplantation models would always be lower than that of the current tumorigenicity test, but rather liked to know the result to evaluate the susceptibility to generate teratomas of the current tumorigenicity test in comparison with other immune tolerance system.

These are prerequisites for performing a series of studies to address the extrapolability of the current tumorigenicity test and we performed a mouse PSC syngeneic transplantation study by subcutaneous administration prior to the pivotal transplantation experiments to verify our assumption that the mPSC syngeneic transplantation model would generate teratomas with a lower cell dose than the hiPSC xeno transplantation model. Indeed, we confirmed that the TPD50 of miPSC-B6 to B6 syngeneic transplantation (Fig. 1B, Supplementary Fig. S1) was calculated to be 11.8 cells (Fig. 1C), which is much lower than that of hiPSC (PFX#9) transplantation into NOG mice to be 215.4 cells and other report with hiPSC.^19^

Based on this test result, we performed a series of syngeneic (Luc-miPSC-B6 to B6 mice or Luc-mESC-129 to 129 mice), allogeneic (Luc-miPSC-B6 to129 mice or Luc-mESC-129 to B6 mice), or xeno (Luc-hiPSC-PFX#9 to NOG mice) tumorigenicity tests using different cell doses and different administration routes.

### Tumorigenicity Tests by Liver Transplantation

**Figure 2.**
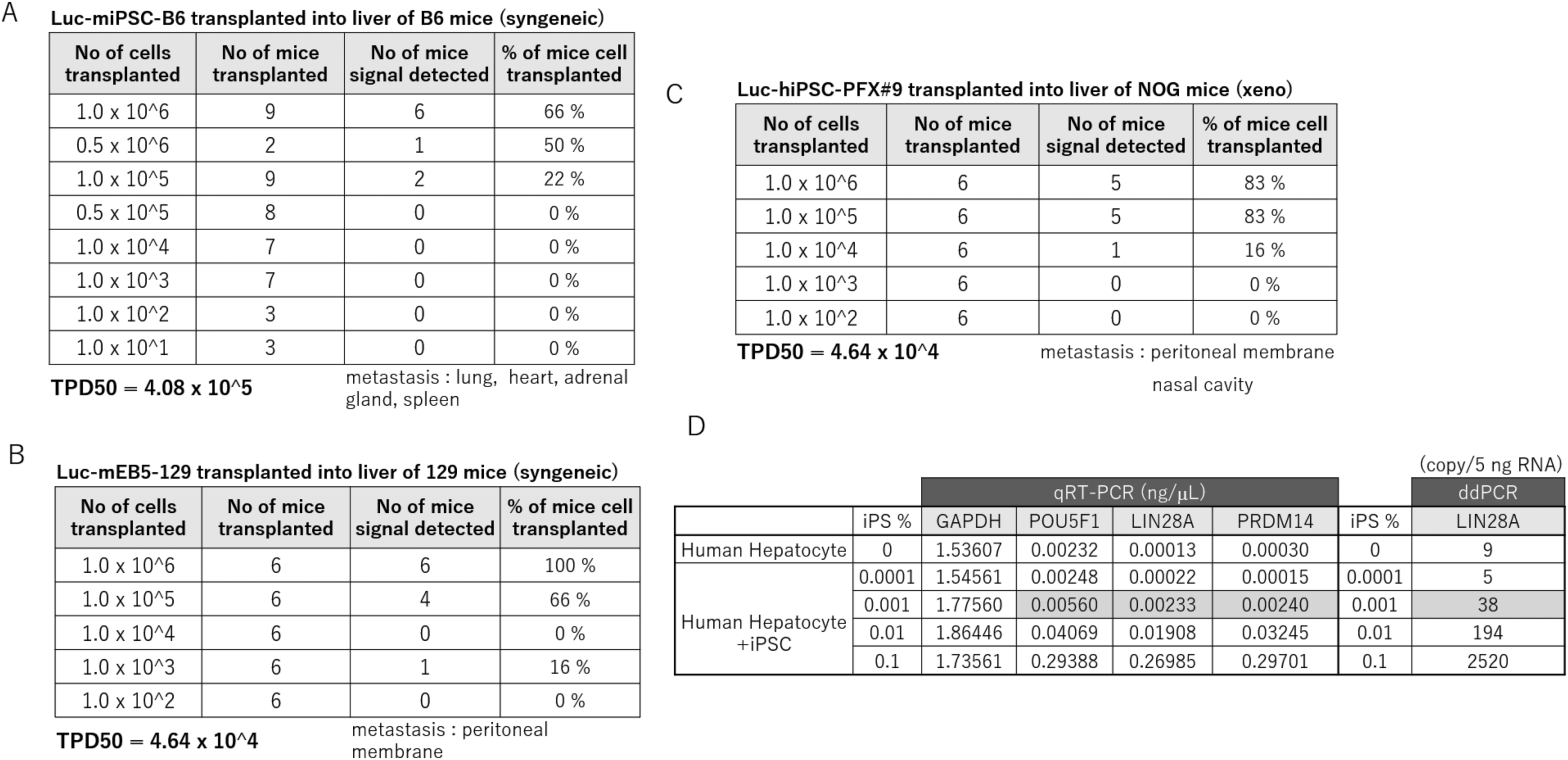
Tumorigenicity tests by liver transplantation. (**A**) The result of tumorigenicity test by transplanting various doses (1 × 10^6^-1 × 10^1^) of Luc-miPSC-B6 cells into B6 mice (syngeneic transplantation). The calculated TPD50 value and the organs where teratomas were found as metastasis are added in the table below. (**B**) The result of tumorigenicity test by transplanting various doses (1 × 10^6^-1 × 10^2^) of Luc-mESC-129 cells into 129 mice (syngeneic-transplantation). The calculated TPD50 value and organs where teratomas were found as metastasis are added in the table below. (**C**) The result of tumorigenicity test by transplanting various doses (1 × 10^6^-10^2^) of Luc-hiPSC-PFX#9 cells into NOG mice (xenotransplantation). The calculated TPD50 value and organs where teratomas were found as metastasis are added in the table below. (**D**) Detection limit of undifferentiated iPSCs in hepatocytes. iPSCs were spiked into hepatocytes at a ratio of 1:10^3^, 10^4^, 10^5^, or 10^6^. Detection of undifferentiated iPSCs in nanogram per milliliter in the mixtures was determined using the primers for POU5F1, LIN28, PRDM14 by qRT-PCR or that in copy number per 5 ng RNA was determined using primers for LIN28A by ddPCR.

The TPD50 value for syngeneic liver transplantation with Luc-miPSC-B6 was 4.08 × 10^5^ cells (Fig. 2A, Supplementary Fig. S2A-1, S2A-2), while that for Luc-mESC-129 was 4.64 × 10^4^ cells (Fig. 2B, Supplementary Fig. S2B-1, S2B-2, S2B-3), and for xeno transplantation with Luc-hiPSC-PFX#9 was 4.64 × 10^4^ cells (Fig. 2C, S2C-1, S2C-2). A series of transplantation studies showed that when the signal from the transplanted cell mass was detected in the liver at 4 weeks, then the cell mass grew to the detectable tumor in the liver and in other organs such as lung, heart, adrenal gland as metastasis in B6 mice, peritoneal membrane in 129 mice, peritoneal membrane, and nasal cavity in NOG mice (Supplementary Fig. S2A-1, S2B-1, S2B-3, S2C-1).

The TPD50 value for syngeneic Luc-miPSC-B6 cell transplantation was higher and that for syngeneic Luc-mESC-129 cell transplantation was lower than that for human iPSCs transplantation, indicating a marked difference in the potential to form a teratoma in the liver between 2 different mouse strains (B6 and 129), and the susceptibility to generate teratoma from transplanted undifferentiated hiPSC is comparable to the B6 and 129 syngeneic mouse model when the immuno-deficient NOG mouse transplantation model is used. The detection limit for the incorporation of undifferentiated hPSCs in hepatocytes by Quantitative RT-PCR(qRT-PCR) or droplet digital PCR (ddPCR) was 0.001%^22^ (Fig. 2D).

### Tumorigenicity Tests by Brain Striatum Transplantation

**Figure 3.**
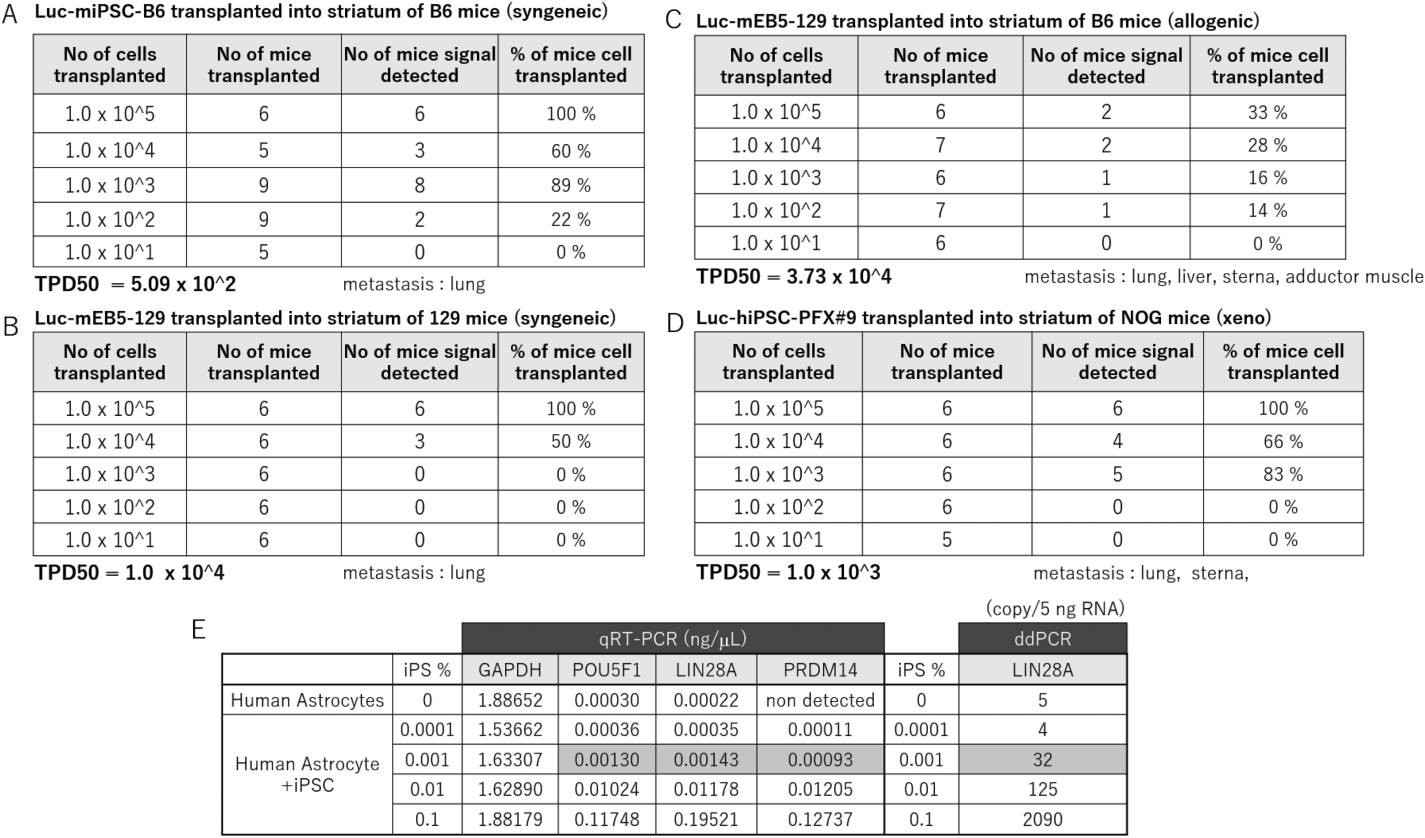
Tumorigenicity tests by brain striatum transplantation. (**A**) The result of tumorigenicity test by transplanting different doses (1 × 10^5^-1 × 10^1^) of Luc-miPSC-B6 cells into B6 mice (syngeneic-transplantation). The calculated TPD50 value and organs where teratomas were found as metastasis are added in below table. (**B**) The result of tumorigenicity test by transplanting different doses (1 × 10^5^-1 × 10^1^) of Luc-mESC-129 cells into129 mice (syngeneic-transplantation). The calculated TPD50 value and organs where teratomas were found as metastasis are added in below table. (**C**) The result of tumorigenicity test by transplanting different doses (1 × 10^5^ – 1 × 10^1^) of Luc-mESC-129 cells into B6 mice (allogeneic transplantation). The calculated TPD50 value and organs where teratomas were found as metastasis are added in the table below. (**D**) The result of tumorigenicity test by transplanting different doses (1 × 10^5^-1 × 10^1^) of Luc-hiPSC-PFX#9 cells into NOG mice (xenotransplantation). The calculated TPD50 value and organs where teratomas were found as metastasis are added in below table. (**E**) Detection limit of undifferentiated iPSCs in astrocytes. iPSCs were spiked into astrocytes at a ratio of 1:10^3^, 10^4^, 10^5^, or 10^6^. Detection of undifferentiated iPSCs in nanogram per milliliter in the mixture was determined using the primers for POU5F1, LIN28, PRDM14 with qRT-PCR or in copy number per 5 ng RNA was determined using primers for LIN28A by ddPCR.

The TPD50 value was 5.09 × 10^2^ cells for syngeneic striatum transplantation of Luc-miPSC-B6 transplanted into B6 mice (Fig. 3A, Supplementary Fig. S3A-1, S3A-2) and 1.0 × 10^4^ cells for Luc-mESC-129 transplanted into 129 mice (Fig. 3B, Supplementary Fig. S3B-1, S3B-2), indicating the presence of variance in teratoma forming potential among the mouse strains. The TPD50 for allogeneic striatum transplantation Luc-mESC-129 to B6 mice was 3.73 × 10^4^ cells (Fig. 3C, Supplementary Fig. S3C-1, S3C-2, S3C-3) and 1.0 × 10^3^ cells for xeno Luc-hiPSC-PFX#9 transplanted in NOG mice (Fig. 3D, Supplementary Fig. S3D-1), suggesting that the current tumorigenicity test of hiPSC using NOG mice would be relevant to the mouse allogeneic counterpart when transplanted into striatum in terms of susceptibility to generate tumor from undifferentiated PSC.

Meanwhile detection limit for the incorporation of undifferentiated iPSCs into neural cells such as astrocytes is 0.001% by qRT-PCR or ddRCR (Fig. 3E).

### Tumorigenicity Tests by Tail Vein Infusion

**Figure 4.**
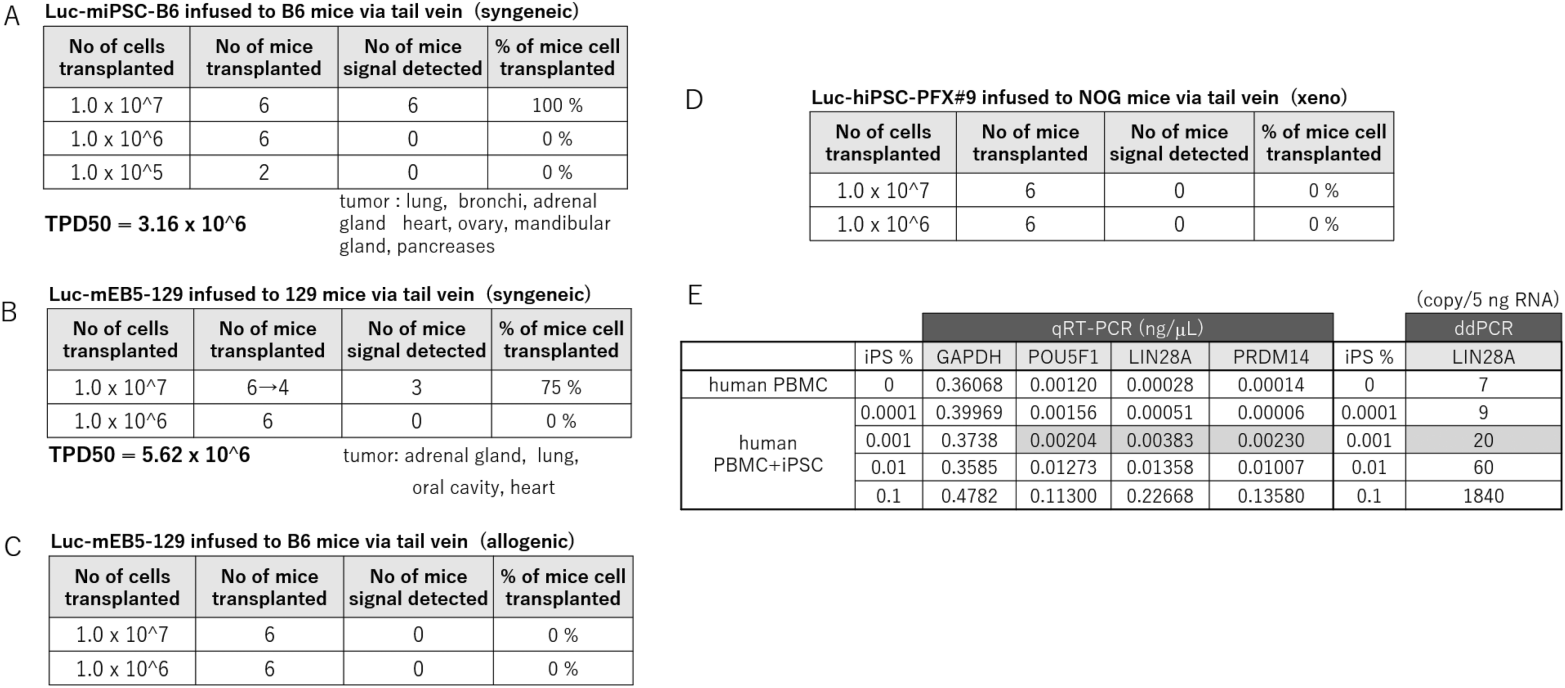
Tumorigenicity tests via tail vein administration. (**A**) The result of tumorigenicity test by infusing various doses (1 × 10^5^-10^7^) of Luc-miPSC-B6 cells into B6 mice (syngeneic-transplantation). The calculated TPD50 value and organs found teratoma as metastasis are added in the table below. (**B**) The result of tumorigenicity test by infusing various doses (1 × 10^6^-10^7^) of Luc-mESC-129 cells into 129 mice (syngeneic-transplantation). The calculated TPD50 value and organs found teratomas as metastasis are added in below table. (**C**) The result of tumorigenicity test by infusing various doses (1 × 10^6^-10^7^) of Luc-mESC-129 into B6 mice (allogeneic transplantation). (**D**) The result of tumorigenicity test by infusing various doses (1 × 10^6^-10^7^) of Luc-hiPS-PFX#9 to NOG mice (xeno transplantation). (**E**) Detection limit of undifferentiated iPSCs PBMC. iPSCs were spiked into PBMC at a ratio of 1:10^3^, 10^4^, 10^5^, or 10^6^. Detection of undifferentiated iPSCs in nanogram per milliliter in the mixture was determined using the primers for POU5F1, LIN28, PRDM14 with qRT-PCR or that in copy number per 5 ng RNA was determined using primers for LIN28A with ddPCR.

The TPD50 value was 3.16 × 10^6^ cells for syngeneic infusion of Luc-miPSC-B6 infused into B6 mice (Fig. 4A, Supplementary Fig. S4A-1, S4A-2) and 5.62 × 10^6^ cells for Luc-129mESC infused into 129 mice (Fig. 4B, Supplementary Fig. S4B-1, S4B-2). Teratomas were found in lung, bronchi, adrenal gland, heart, ovary, mandibular gland and pancreas in B6 mice, and in the lung adrenal gland, heart, ovary, and oral cavity in 129 mice. However, no teratoma formation was observed upon allogeneic infusion of Luc-129mESC (1 × 10^6-7^ cells) into B6 mice (Fig. 4C), or for xeno infusion of Luc-hiPSC-PFX#9 (1 × 10^6-7^ cells) into NOG mice (Fig. 4D).

The TPD50 values for syngeneic cell transplantation with miPSC-B6 or mESC-129 showed no major difference between 2 strains, suggesting adhesive property and potential to form teratomas of B6 mPSC and 129 mPSC are comparable in the organs with abundant blood infusion. It should be noted that no teratoma or tumor was observed after allogeneic transplantation of 1 × 10^7^ mESC-129 cells into B6 mice or xeno transplantation of 1 × 10^7^ hiPSC-PFX#9 into NOG mice, suggesting that the result of current tumorigenicity test using NOG mice via mouse tail vein infusion could provide some idea of the number of hiPSC-derived cells that would not form teratomas from undifferentiated cells in the PSC-derived blood products, if the syngeneic or allogeneic mouse model recapitulates human autologous or allogeneic transfusion. The detection limit for the incorporation of undifferentiated iPSCs in PBMC is 0.001% by qRT-PCR or ddRCR (Fig. 4E).

A summary of the incidence of teratomas by route of administration, observation period, and outcome is shown in Table 1.

**Table 1.** A summary of the incidence of teratomas by route of administration, observation period, and outcome.

Transplanation organ/route	Cell line	Recipient mouse	Minutes. dose (cells) generated teratma	Week last tertaoma was reporeted	Monitoring period (w)	TPD50
sc with matrigel	Luc-miPSC-B6	B6	1 × 10^1	8	12	1.18 × 10^1^
sc with matrigel	Luc-hiPSC	NOG	1 × 10^2	7	12	2.15 × 10^2^
Liver	Luc-miPSC-B6	B6	1 × 10^5	2	12	4.08 × 10^5^
Liver	Luc-mESC-129	129	1 × 10^3	4	12	4.64 × 10^4^
Liver	Luc-hiPSC	NOG	1 × 10^4	2	12	4.64 × 10^4^
Striatum	Luc-miPSC-B6	B6	1 × 10^2	6	27	5.09 × 10^2^
Striatum	Luc-mESC-129	129	1 × 10^4	4	38	1.0 x 10^4^
Striatum	Luc-mESC-129	B6	1 × 10^2	6	16	3.73 × 10^4^
Striatum	Luc-hiPSC	NOG	1 × 10^3	8	49	1.0 x 10^3^
i.v.	Luc-miPSC-B6	B6	1 × 10^7	4	14	3.16 × 10^6^
i.v.	Luc-mESC-129	129	1 × 10^7	4	36	5.62 × 10^6^
i.v.	Luc-mESC-129	B6	*No teratoma formation	No teratoma formation	36	Not applicable
i.v.	Luc-hiPSC	NOG	*No teratoma formation	No teratoma formation	36	Not applicable

*Infused 1 × 10^7^ cells.

Abbreviation: sc: subcutaneous, i.v.: intravenous pink B6 origin, blue: 129 origin, orange: NOG mouse, green:hiPSC

### Morphology of Teratomas from Syngeneic-, Allogeneic-, or Xenotransplantation

Teratomas generated from undifferentiated B6-iPSC and h-iPSC (PFX#9) consist of well-differentiated cells of 3 germ layers origin when transplanted subcutaneously (Supplementary Fig. S5A, S5B), and those generated from undifferentiated B6-iPSC and 129-ESC consist mainly of not well-differentiated neural cells, which contrasted with those generated from undifferentiated h-iPSC (PFX#9) (Supplementary Fig. S5C, S5D, S5E). It is interesting to note that both undifferentiated B6-iPSC and 129-ESC generated teratoma-like tumors with a clear boundary consisting of dominantly immature neural cells forming a rosette, whereas h-iPSC (PFX#9) generated those consisting of differentiated neural cells including GFAP + glial cells without a rosette (Supplementary Fig. S5F, S5G, S5H, S5I), suggesting that the composition of teratomas in 3 germ layers is influenced by the characteristic feature of the microenvironment where undifferentiated cells are transplanted, and the state of pluripotency of human PSC (primed type) and mouse PSC (naïve type).

## Discussions and Conclusion

Several cell labeling methods^29-31^ were investigated prior to conducing a series of mouse transplantation experiments. Among them, the Akaluc system^32^ and the luciferase system^33^ were extensively studied for signal strength and durability. We selected the luciferase system in this study based on the reports of no signal bias depending on the type of organ, no background signal from living cells, no toxic effect on rodents after administration of substrate, and no induction of dermatitis after administration of substrate^33^ and found that the detection of the cell mass consisting of 300 cells by the luciferase system would be adequate to conduct a series of tumorigenicity tests and biodistribution experiment after cell transplantation.

We questioned the extrapolability of the current tumorigenicity test using immunodeficient mice by comparing the test result with that of syngeneic or “modulated” allogeneic mouse models. For this attempt, we needed a number of prerequisites, such as that a syngeneic or allogeneic transplantation mouse model would closely recapitulate human autologous or allogeneic transplantation, or that the resistance to mechanical stress and survival of hiPSC and mPSC after needle injection would be considered to be comparable. However, we believe that the challenge to study the extrapolability of the current tumorigenicity test is important to ensure the safety of the PSC-based cell therapy.

During the course of experiment, we found that there was a marked difference in the TPD50 values of B6 and 129 syngeneic transplants in the striatum injection despite the same HLA background. This result may represent a difference in the survival potential of cells after injection into the striatum and its microenvironment to support the ectopically engrafted cells. Despite these assumptions, TPD50 values from syngeneic transplantation models were found to be lower than those from relevant “modulated” allogeneic transplantation models, and the TPD50 values from xenotransplantation models are comparable to those from syngeneic or “modulated” allogeneic transplantation models. These results suggest that the result of the current tumorigenicity test using NOG mice can be used to infer the occurrence of teratoma, not to say tumor, in hPSC-derived cell therapy, since we only use undifferentiated cell as positive control cells and do not know of any other relevant tumorigenic cell to generate abnormal cell mass. If we get negative result from the current tumorigenicity test, we could say that the tumorigenicity of the final product is not detected under the current tumorigenicity test and would not stop the application process for clinic with this test result, while if we get positive result, we should stop the project for clinic until we solve the problem. Of course, it is obvious that the accumulation of data by conducting a mouse study with a large number of mice of different strains and different hiPSC clones would strengthen this statement from a risk management perspective.

Based on above argument, we suggest that the likelihood of teratoma formation from undifferentiated cells that possibly contained in hPSC-derived hepatocytes would increase if 1 × 10^8^ cells [(the lowest dose to generate teratoma from syngeneic transplantation in 129 mice; 1 × 10^3^ cells) × (detection limit for undifferentiated cells by PCR is 0.001%; 1 × 10^5^)] or more autologous or possibly allogeneic hPSCs-derived hepatocytes are transplanted into the liver, provided that no detectable inclusion of undifferentiated cells in the final product is reported by qRT-PCR test.

We suggest that the likelihood of teratoma formation from undifferentiated cells that may be present in hPSC-derived neural cells would be increased if 1 × 10^7^ [(the lowest dose to produce teratoma in B6 mice; 1 × 10^2^ cells) × (detection limit for immature hPSCs is 0.001% by PCR; 1 × 10^5^)] or more autologous or allogeneic hPSC-derived neural cells are transplanted via the striatum, provided that no detectable inclusion of undifferentiated cells in the final product is reported by qRT-PCR testing.

We suggest that the likelihood of teratoma formation from undifferentiated cells possibly contained in hPSC-derived blood cells would increase if 2 × 10^14^ cells [(highest dose without teratoma incidence in B6 or 129 mice: 1 × 10^6^) × (detection limit for undifferentiated cell inclusion in PBMC is 0. 001% by qRT-PCR: 1 × 10^−5^) × (human/mouse weight ratio: 2 × 10^3^)] or more autologous hPSC-derived blood cells are infused and 2 × 10^15^ cells [(the highest dose tested without incident in B6 or 129 mice: 1 × 10^7^) × (detection limit for undifferentiated cells inclusion in PBMC is 0. 001% by qRT-PCR: 1 × 10^−5^) × (human/mouse weight ratio: 2 × 10^3^)] or more allogeneic hPSC-derived blood cells are infused, provided that no detectable inclusion of undifferentiated cells in the final product is reported by qRT-PCR. Human undifferentiated PSCs are adherent cells and prone to undergo apoptosis in the culture condition that supports for suspension cells, therefore, the likelihood of teratoma formation from undifferentiated cells that we estimated for blood cells would be fairly supported from a risk management perspective.

Further studies to bridge the gap between murine transplantation models and human clinical outcomes would provide clearer insight into the extrapolability of the current tumorigenicity test and ensure the safety of PSC-based cell therapy.

## Supplementary Material

Supplementary material is available at *Stem Cells Translational Medicine* online.

szae019_suppl_Supplementary_Material

## Data Availability

The data underlying this article will be shared on reasonable request to the corresponding author.
