# Supplementary material for: Study on the Extrapolability of Current Tumorgenicity Test With Mice by Comparing the Syngeneic or Allogeneic Mouse Transplantation Model
Source: Stem Cells Transl Med. 2024 Jun 10;13(6):572–81. doi: 10.1093/stcltm/szae019 (PMC11165165; doi:10.1093/stcltm/szae019)

Fig. S1

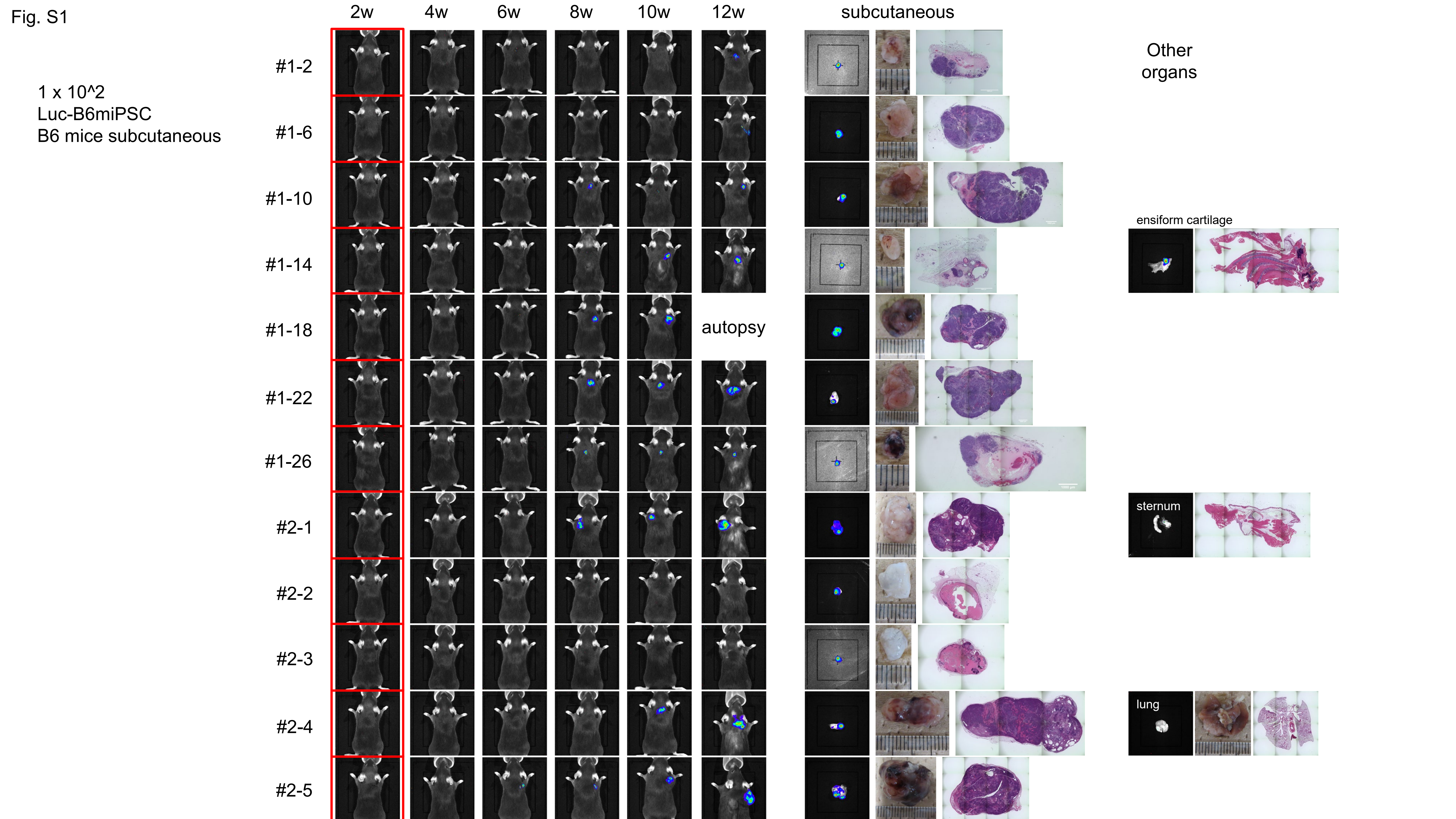
1 x 10^2
Luc-B6miPSC
B6 mice subcutaneous
2w
4w
6w
8w
10w
12w
subcutaneous
Other organs
#1-2
#1-6
#1-10
#1-14
#1-18
#1-22
#1-26
#2-1
#2-2
#2-3
#2-4
#2-5
autopsy
ensiform cartilage
sternum
lung

Fig. S2A-1

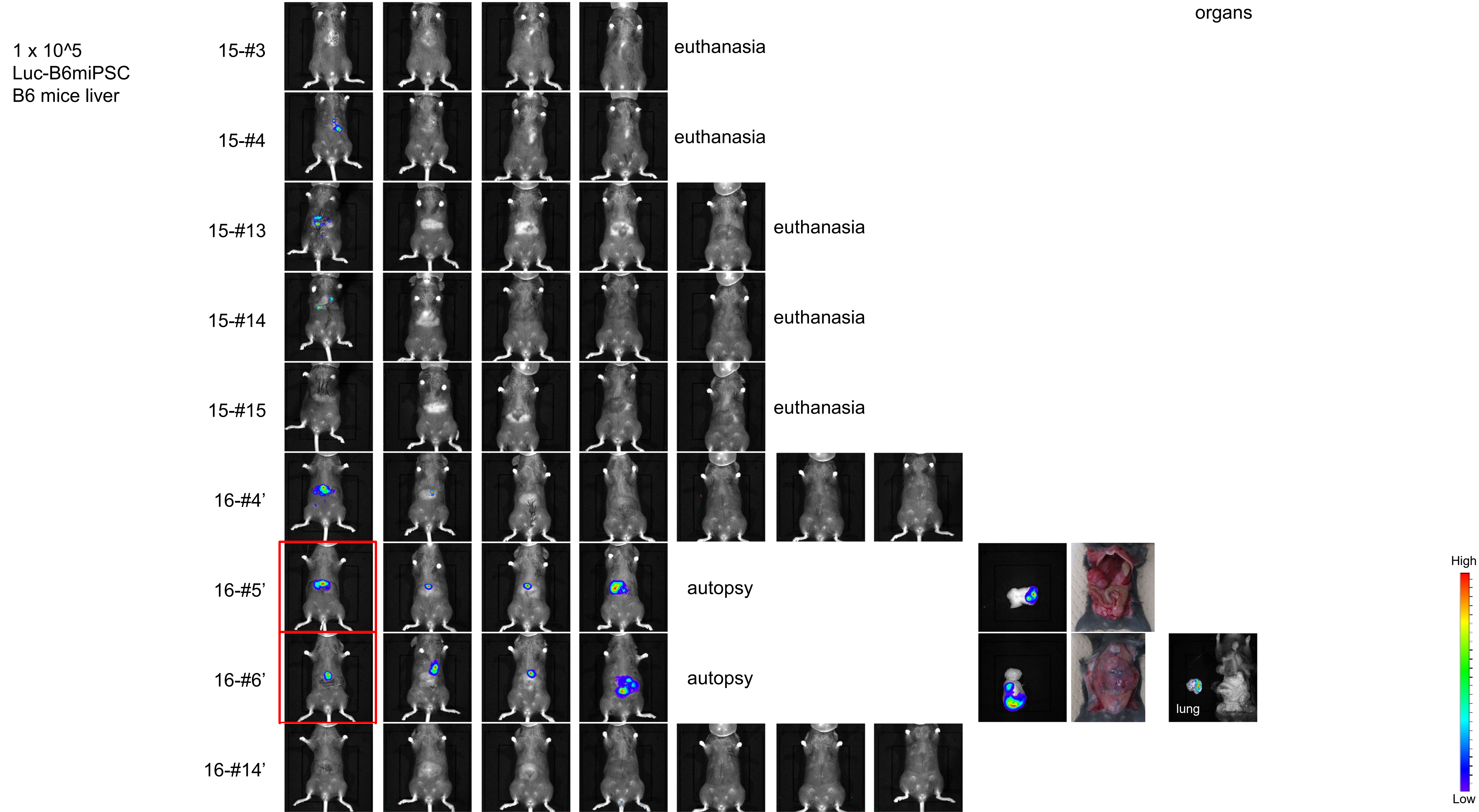
1 x 10^5
Luc-B6miPSC
B6 mice liver
60 min
2w
4w
6w
8w
10w
12w
liver
Other organs
15-#3
euthanasia
15-#4
euthanasia
15-#13
euthanasia
15-#14
euthanasia
15-#15
euthanasia
16-#4’
16-#5’
autopsy
16-#6’
autopsy
lung
16-#14’
High
Low

Fig. S2A-2

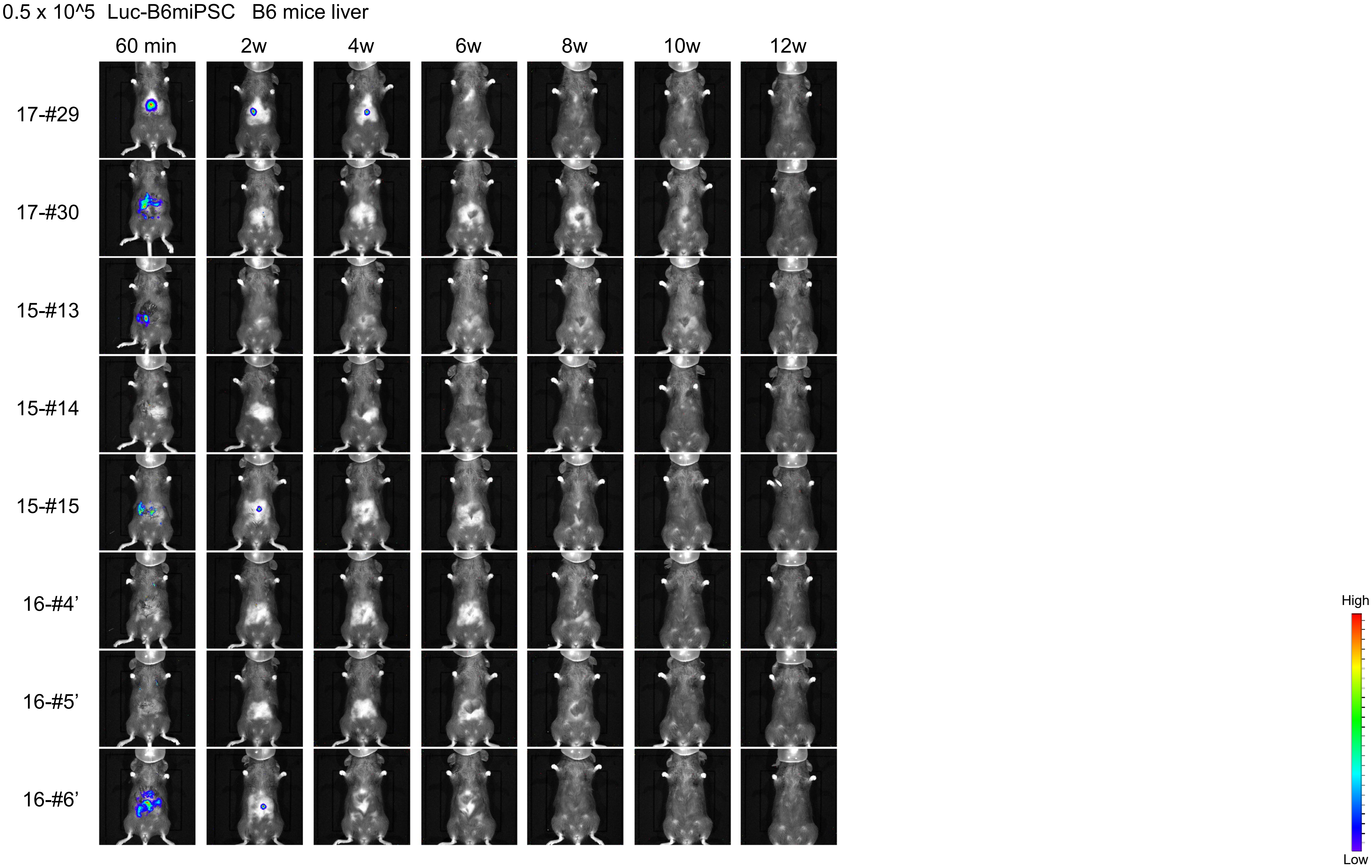

0.5 x 10^5 Luc-B6miPSC B6 mice liver
60 min
2w
4w
6w
8w
10w
12w
17-#29
17-#30
15-#13
15-#14
15-#15
16-#4’
16-#5’
16-#6’
High
Low

Fig. S2B-1

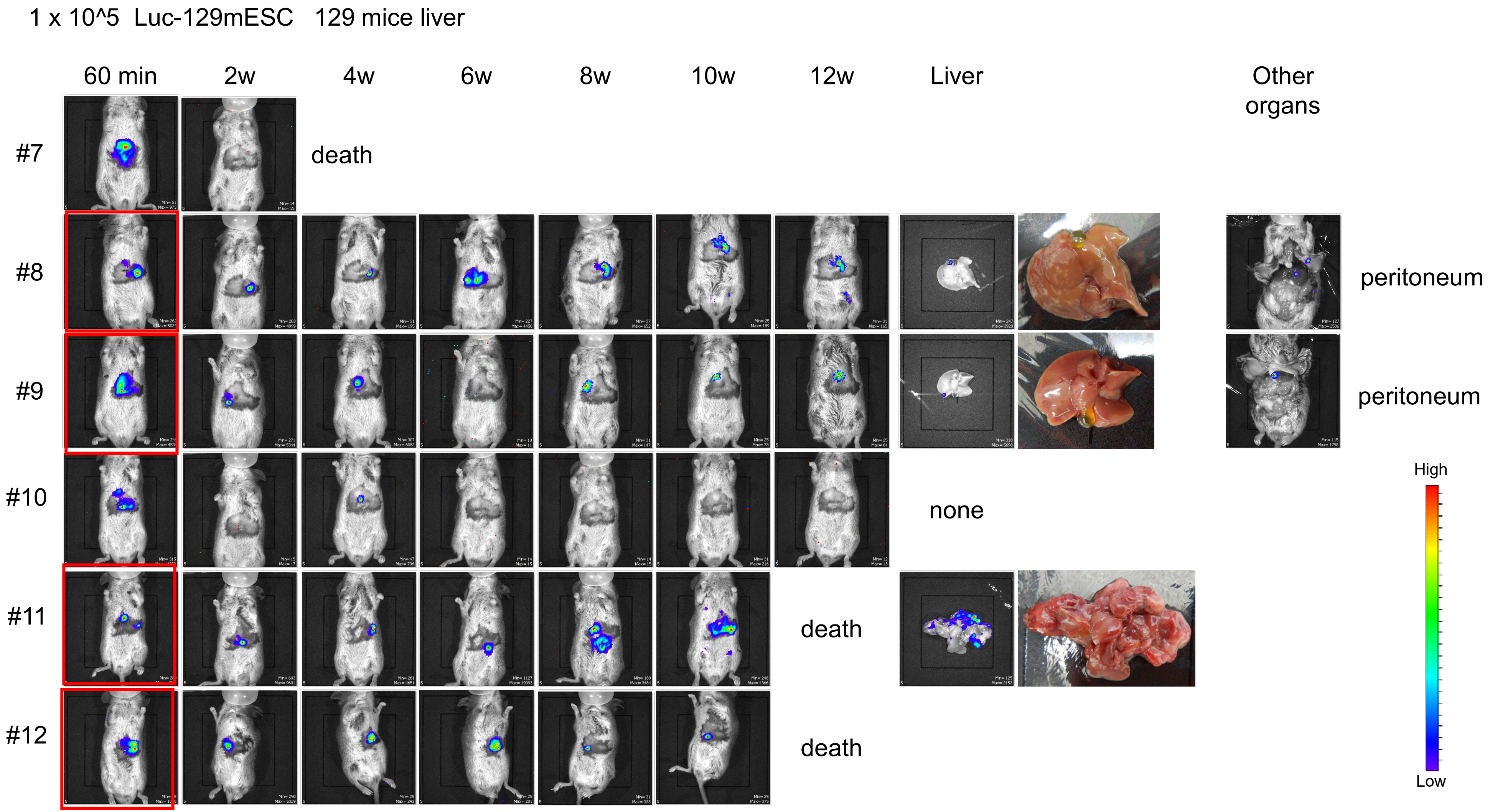
1 x 10^5 Luc-129mESC 129 mice liver
60 min
2w
4w
6w
8w
10w
12w
Liver
Other organs
#7
death
#8
peritoneum
#9
peritoneum
#10
none
#11
death
#12
death
High
Low

Fig. S2B-2

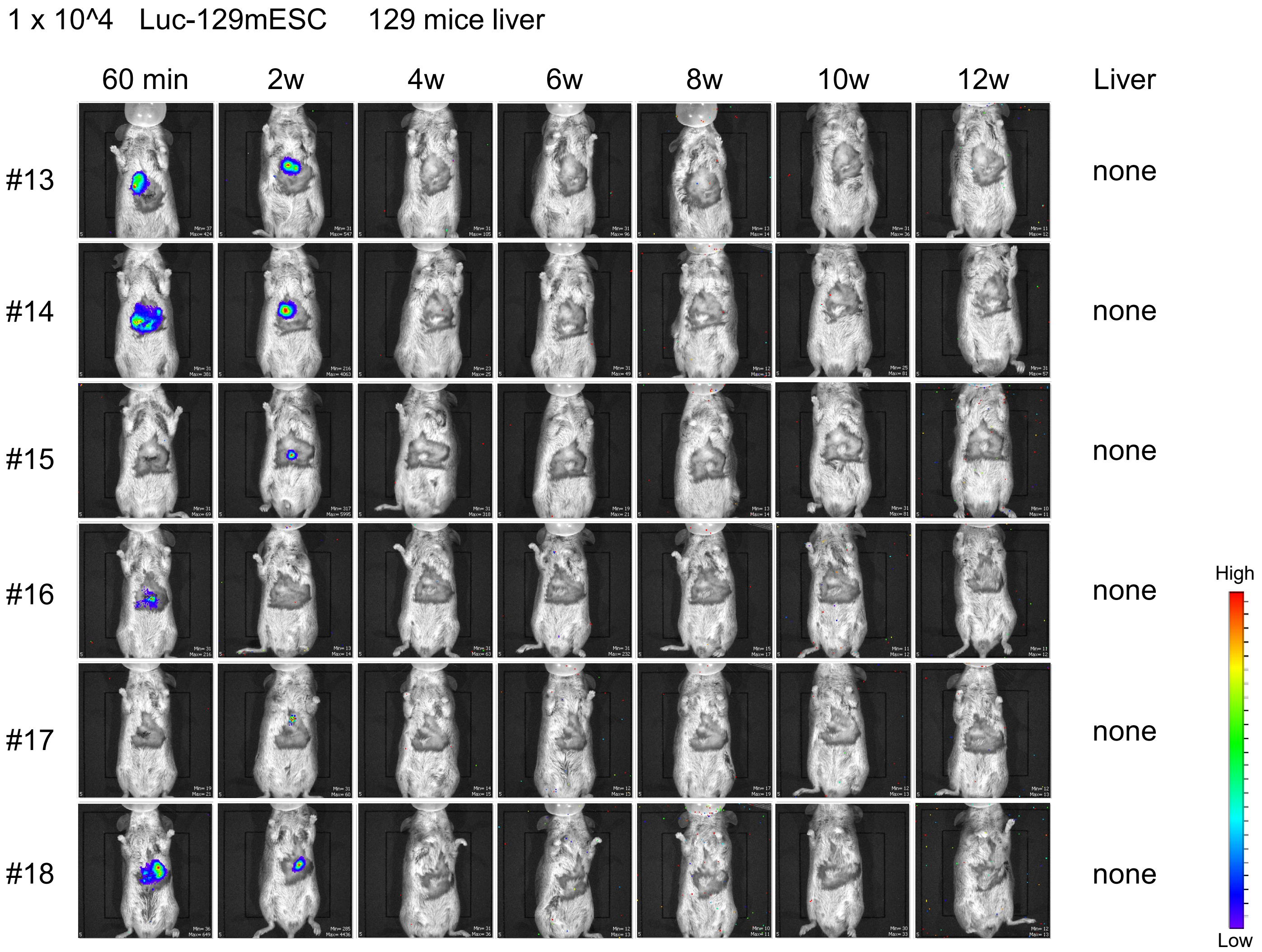
1 x 10^4 Luc-129mESC 129 mice liver
60 min
2w
4w
6w
8w
10w
12w
Liver
#13
#14
#15
#16
#17
#18
none
none
none
none
none
none
High
Low

Fig. S2B-3

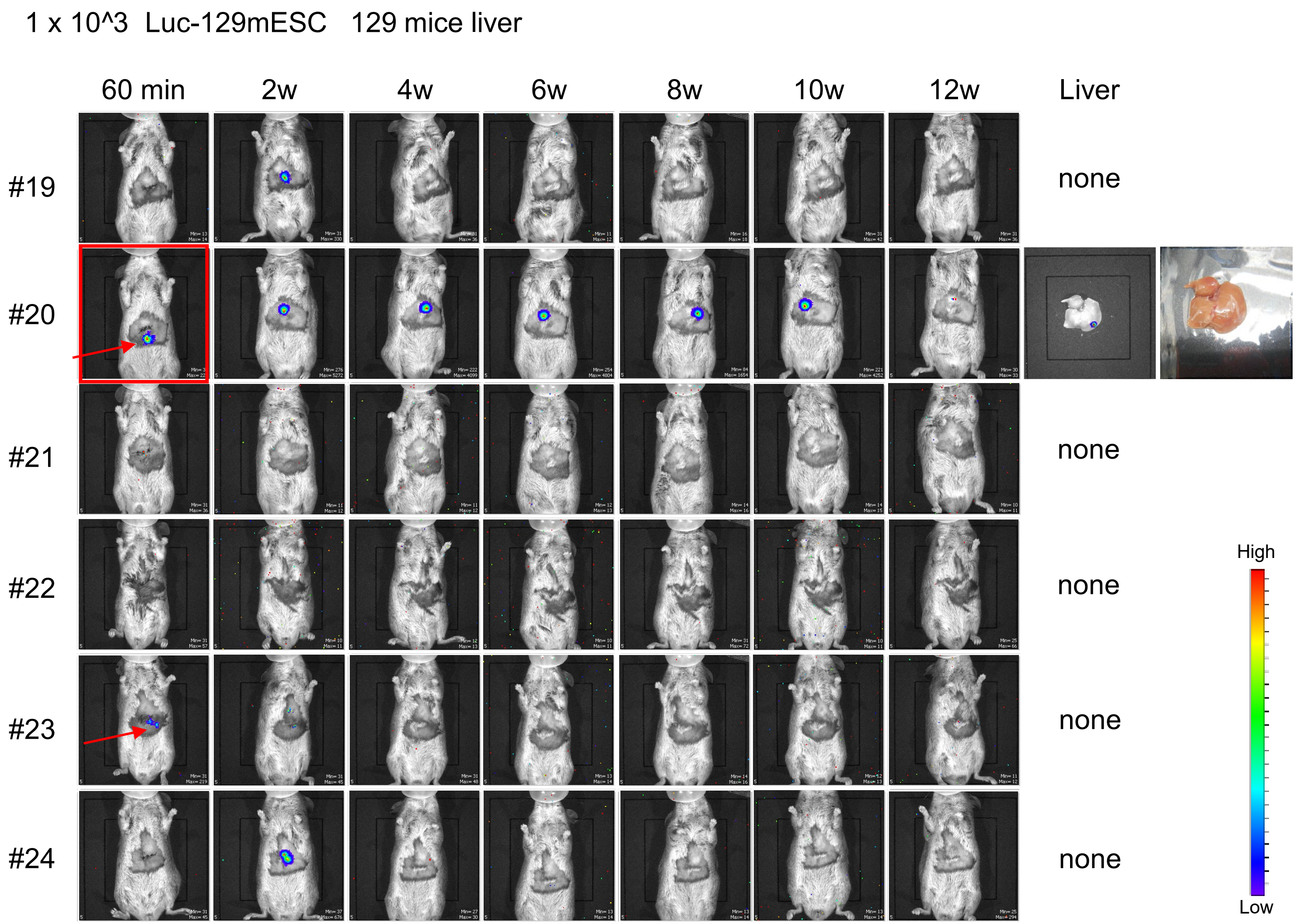
1 x 10^3 Luc-129mESC 129 mice liver
60 min
2w
4w
6w
8w
10w
12w
Liver
#19
#20
#21
#22
#23
#24
none
none
none
none
none
High
Low

Fig. S2C-1.

1 x 10^4 Luc-hiPSC (PFX#9) NOG mice liver

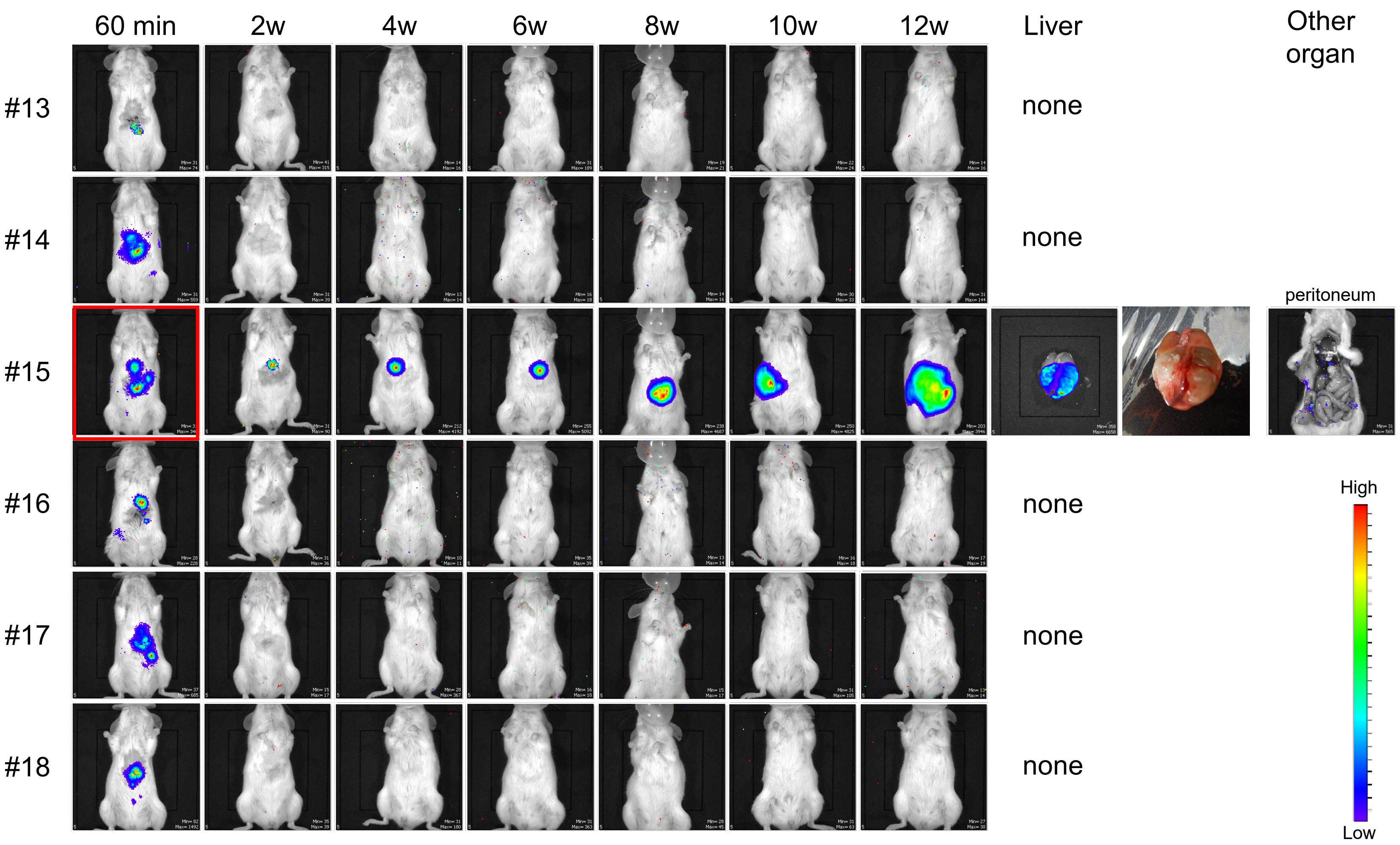

Fig. S2C-2.

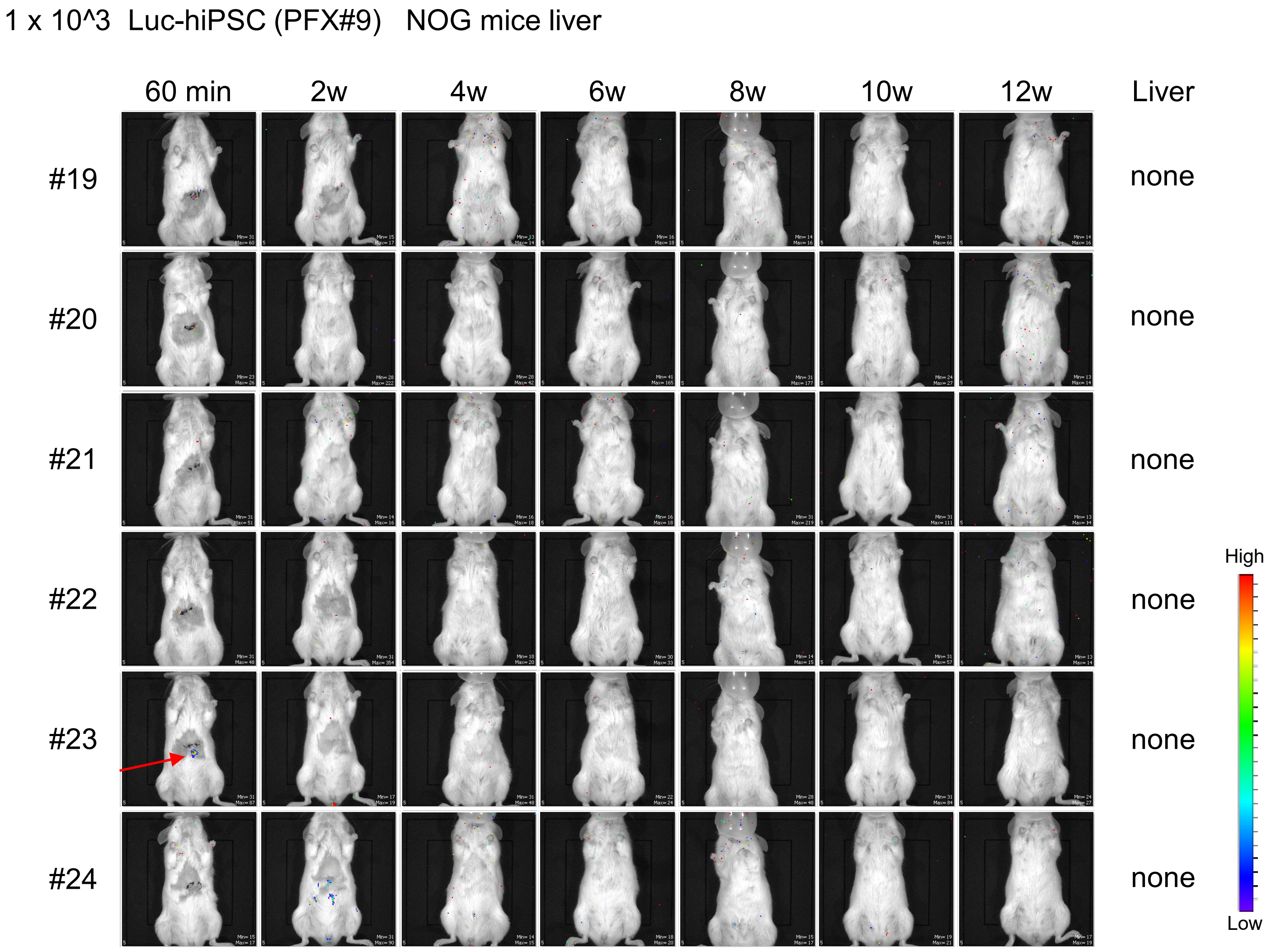
1 x 10^3 Luc-hiPSC (PFX#9) NOG mice liver
60 min
2w
4w
6w
8w
10w
12w
Liver
#19
none
#20
none
#21
none
#22
none
#23
none
#24
none
High
Low

Fig. S3A-1.

1 x 10^3 Luc-B6miPSC B6 mice brain

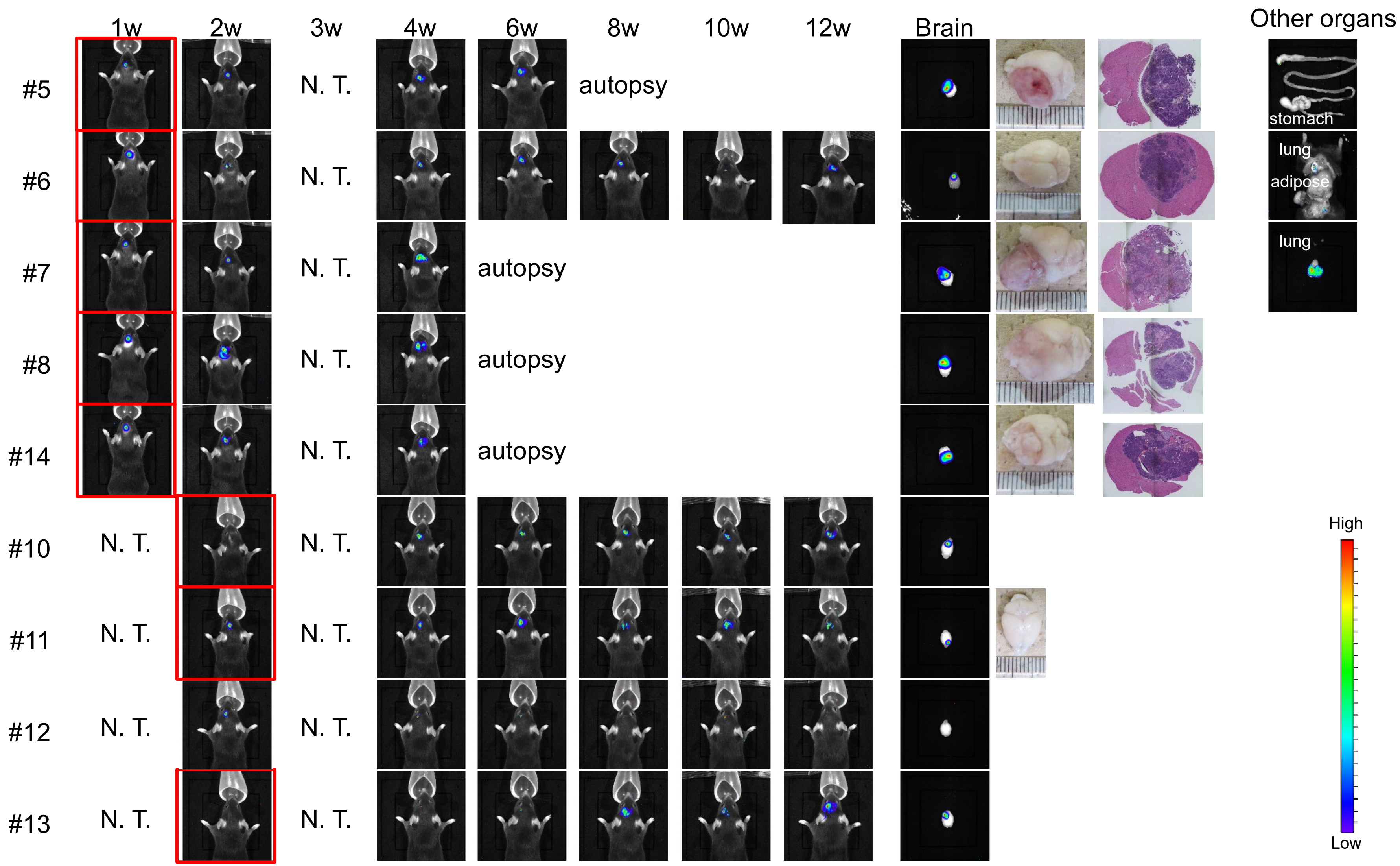

Fig. S3A-2.

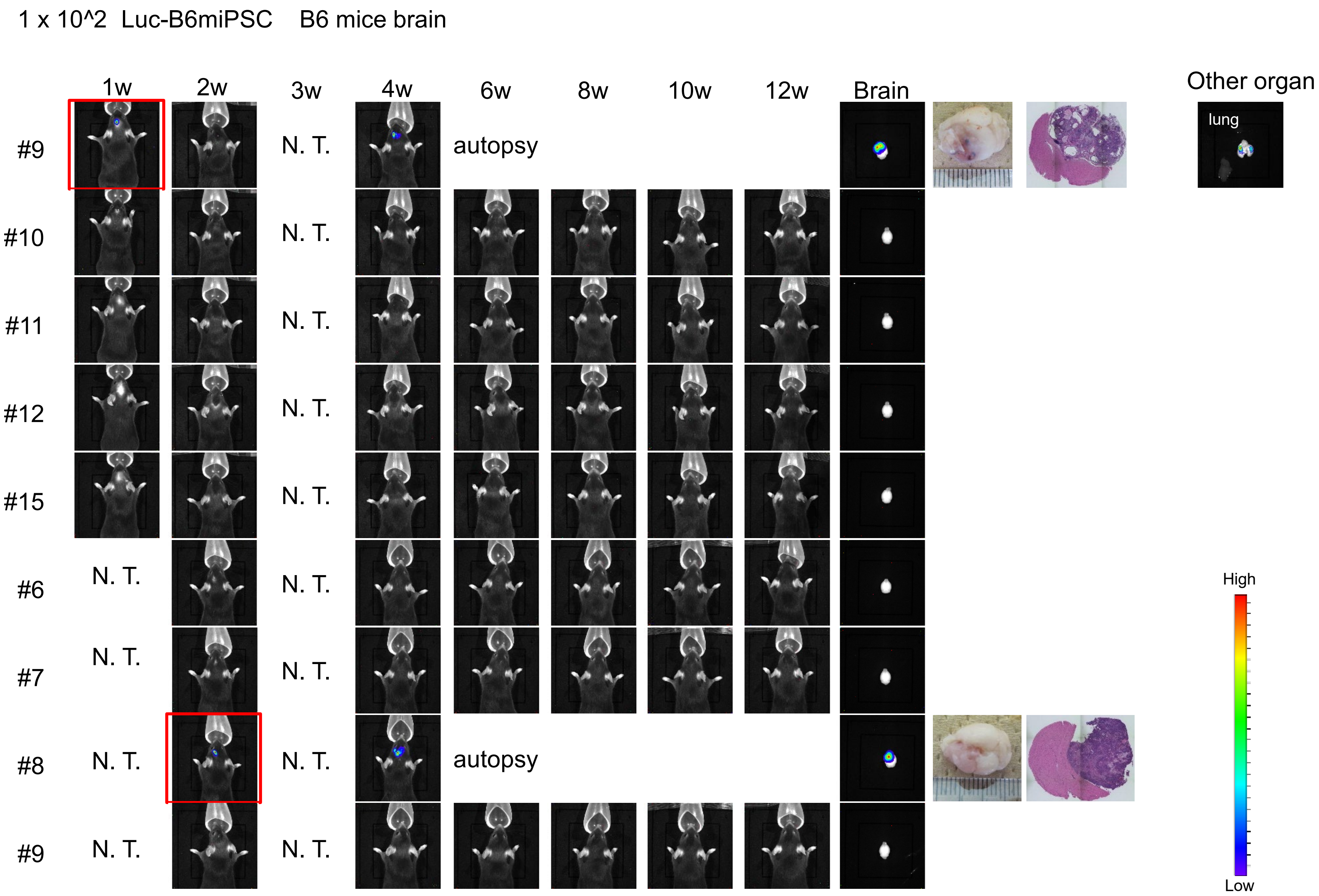
1 x 10^2 Luc-B6miPSC B6 mice brain
1w
2w
3w
4w
6w
8w
10w
12w
Brain
Other organ
#9
#10
#11
#12
#15
#6
#7
#8
#9
N. T.
autopsy
lung
High
Low

Fig S3B-1.

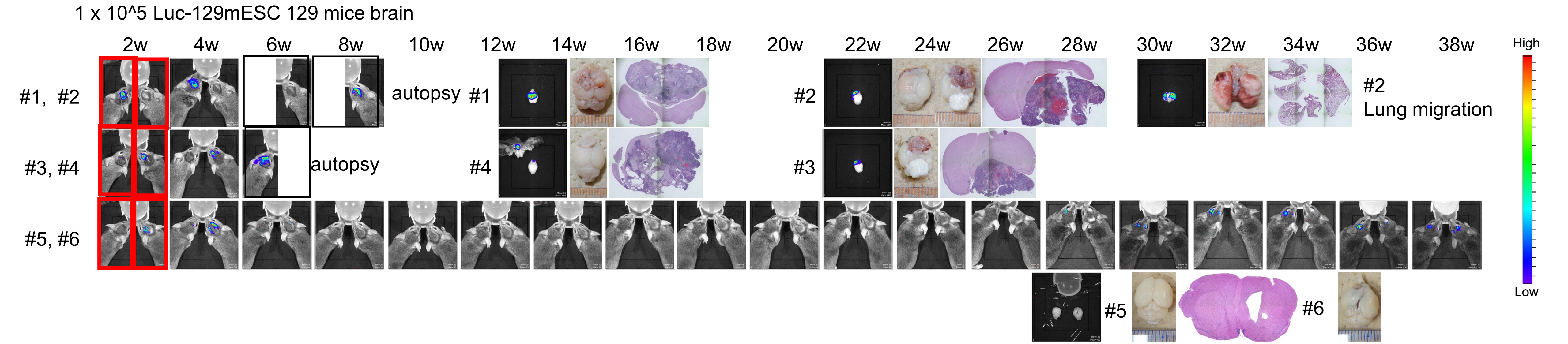

Fig S3B-2.

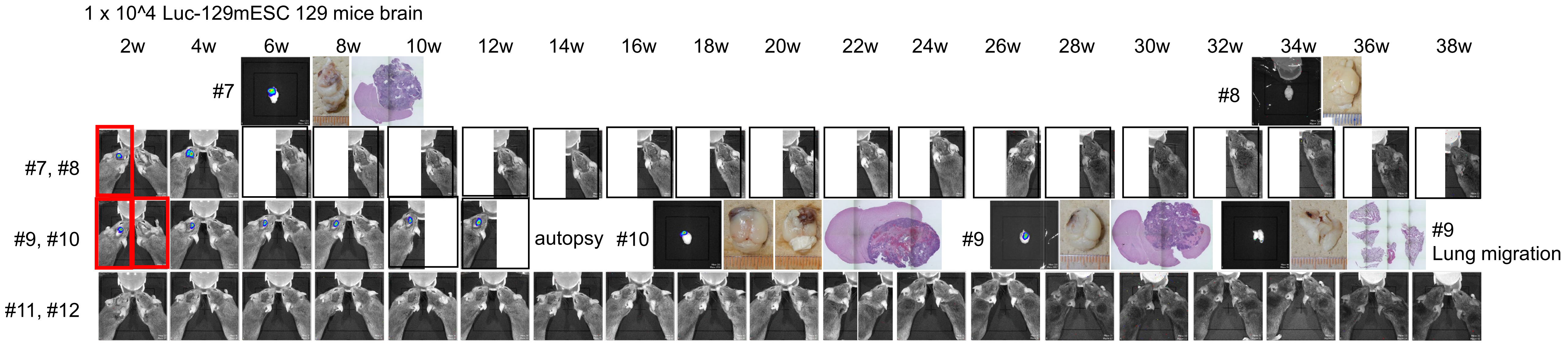

Fig. S3C-1.

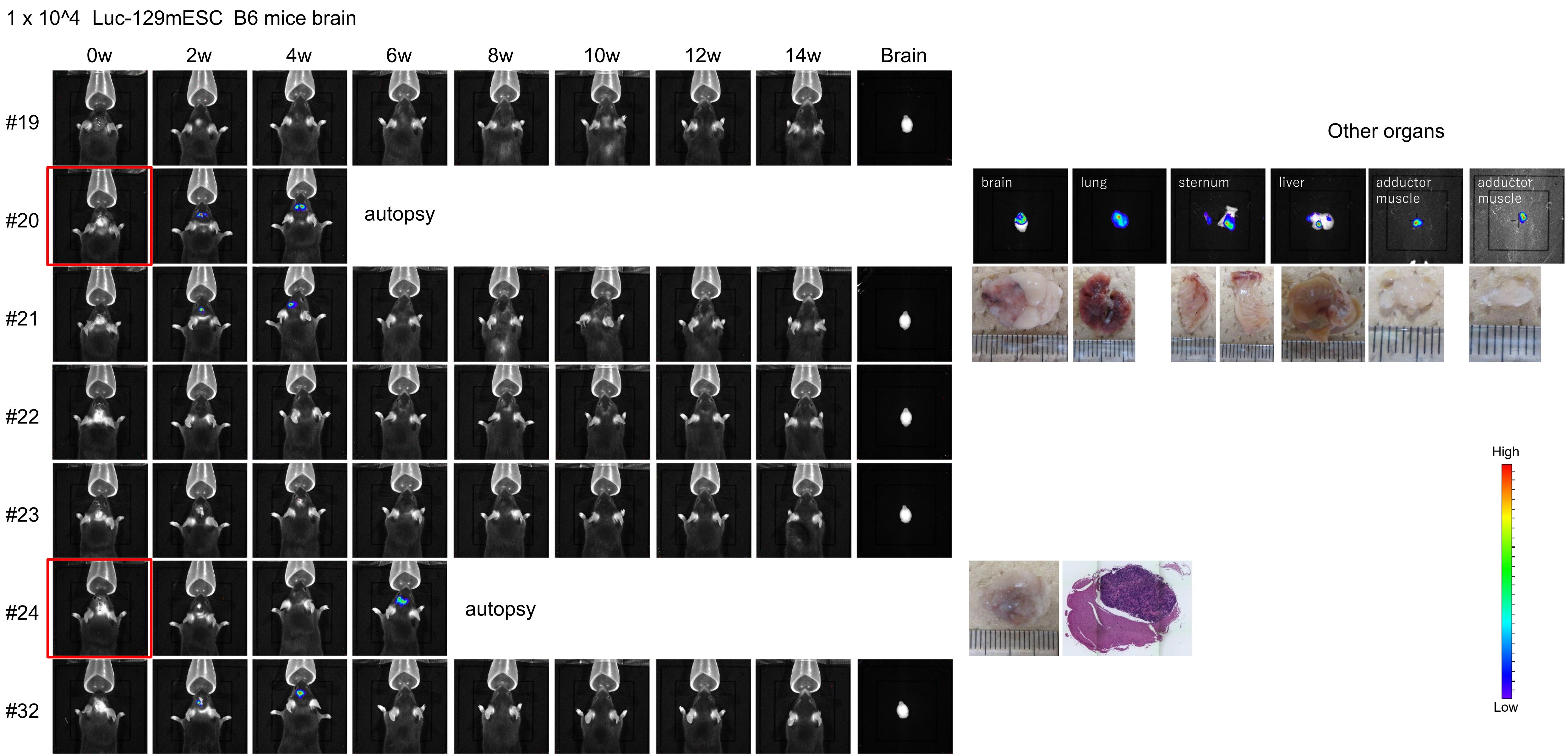
1 x 10^4 Luc-129mESC B6 mice brain
0w
2w
4w
6w
8w
10w
12w
14w
Brain
#19
#20
#21
#22
#23
#24
#32
autopsy
autopsy
Other organs
brain
lung
sternum
liver
adductor muscle
adductor muscle
High
Low

Fig. S3C-2

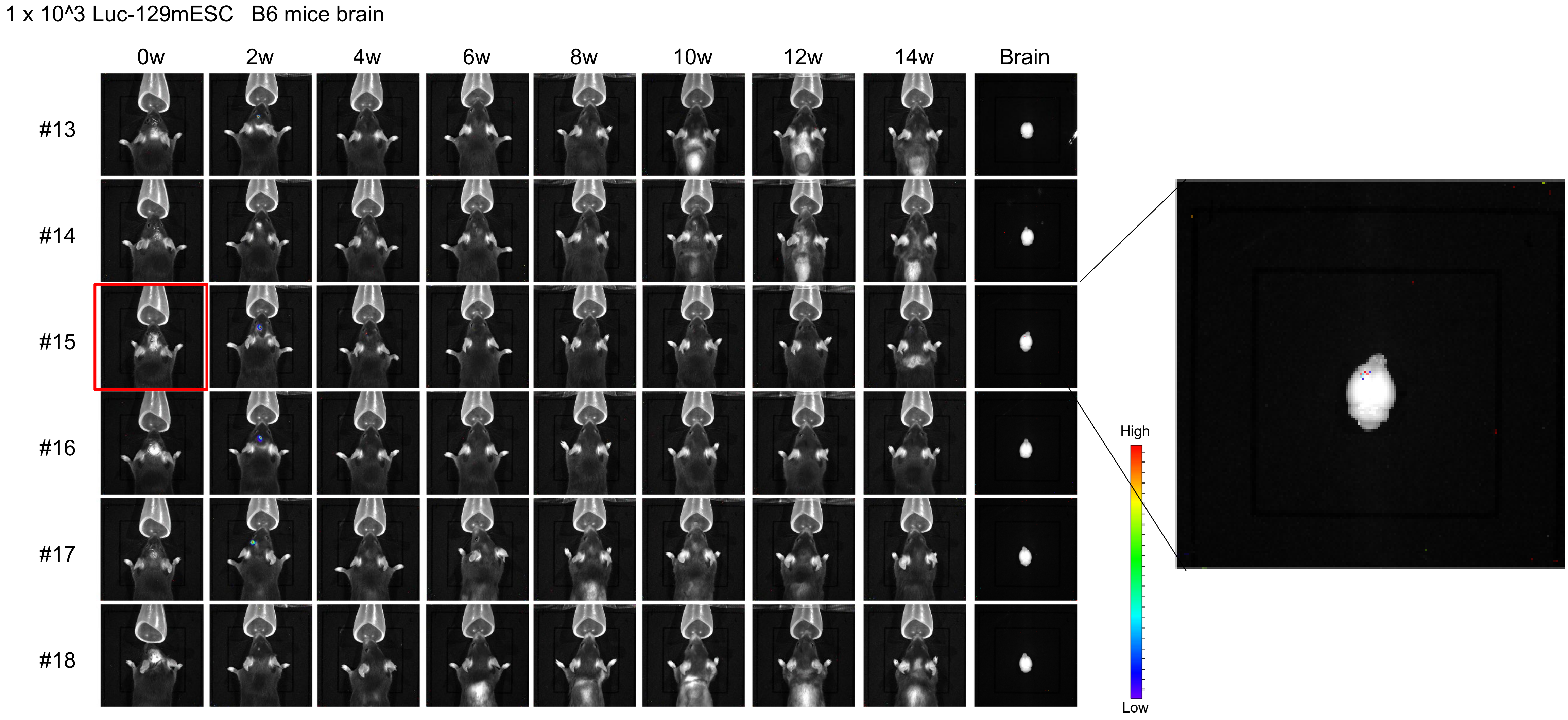
1 x 10^3 Luc-129mESC   B6 mice brain
0w
2w
4w
6w
8w
10w
12w
14w
Brain
#13
#14
#15
#16
#17
#18
High
Low

Fig. S3C-3.

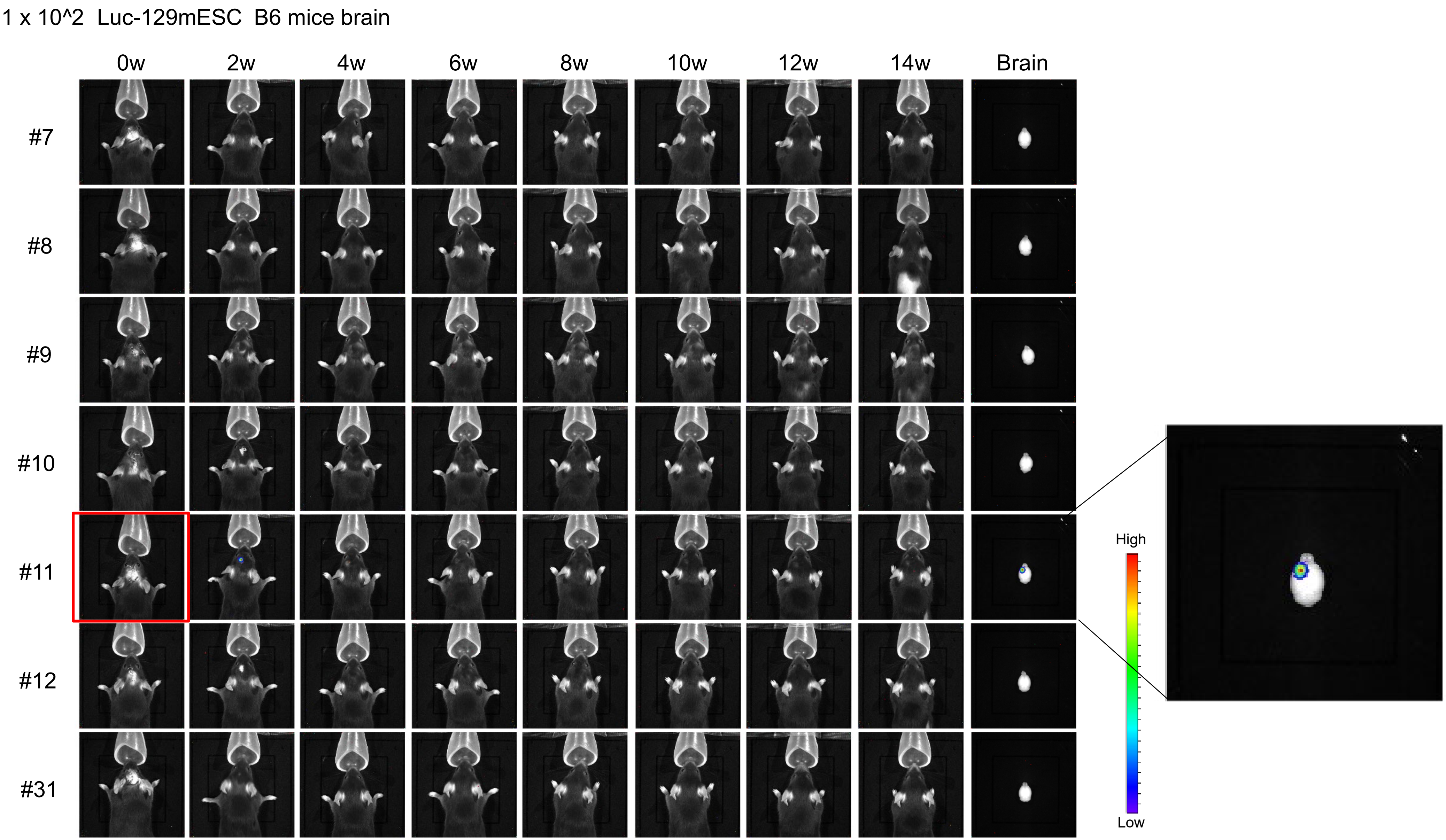
1 x 10^2 Luc-129mESC B6 mice brain
0w
2w
4w
6w
8w
10w
12w
14w
Brain
#7
#8
#9
#10
#11
#12
#31
High
Low

Fig. S3D-1

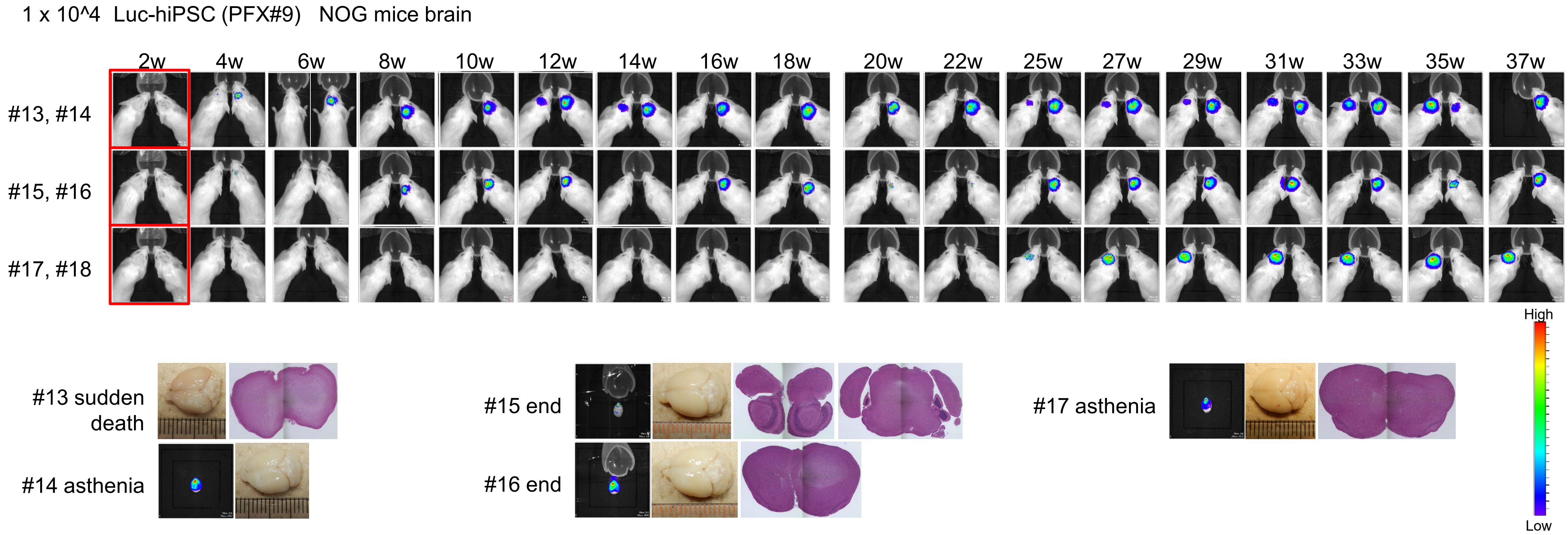
1 x 10^4 Luc-hiPSC (PFX#9) NOG mice brain
2w
4w
6w
8w
10w
12w
14w
16w
18w
20w
22w
25w
27w
29w
31w
33w
35w
37w
#13, #14
#15, #16
#17, #18
High
Low
#13 sudden death
#14 asthenia
#15 end
#16 end
#17 asthenia

Fig. S4A-1.

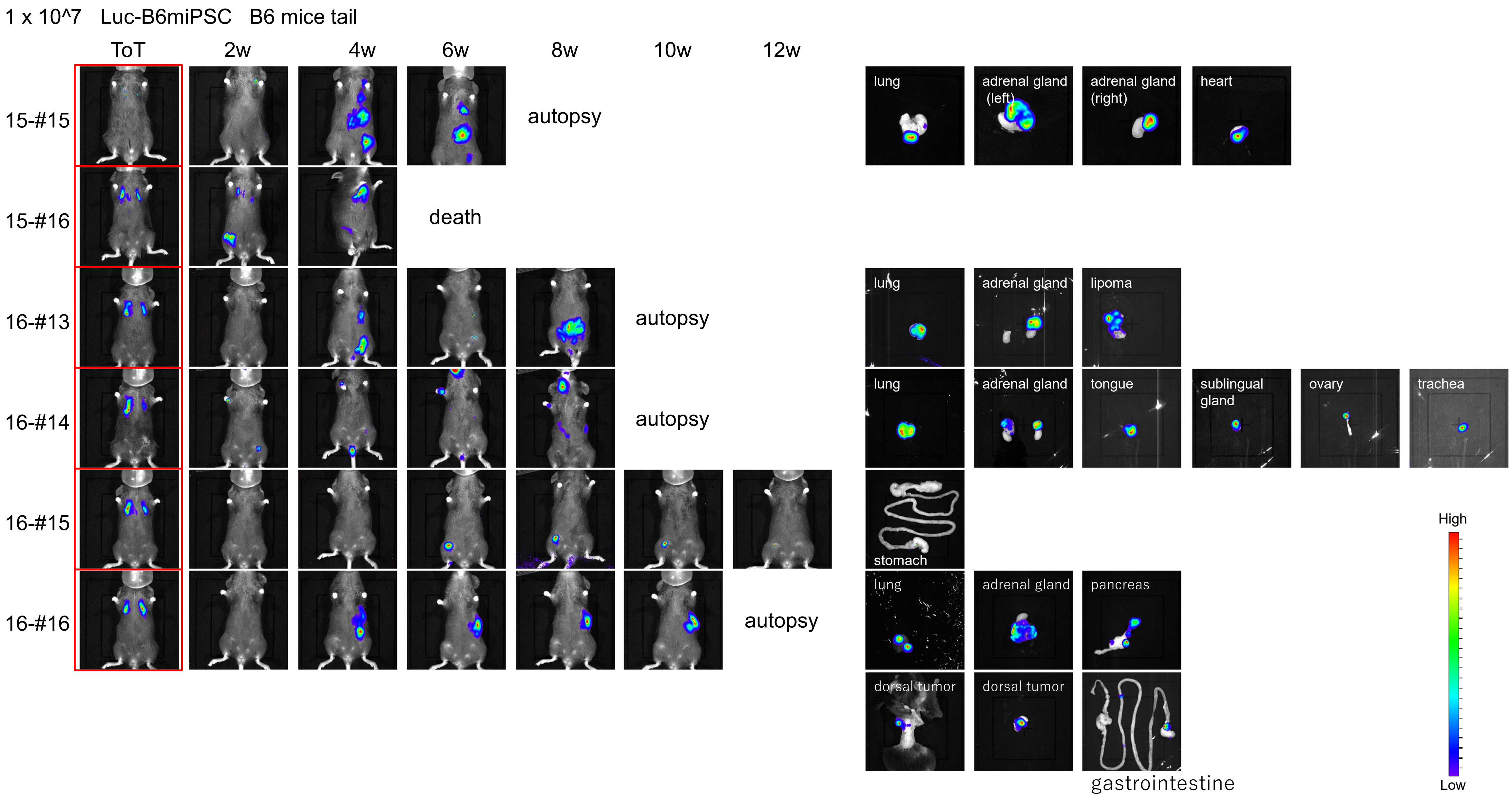
1 x 10^7 Luc-B6miPSC B6 mice tail
ToT
2w
4w
6w
8w
10w
12w
15-#15
autopsy
lung
adrenal gland (left)
adrenal gland (right)
heart
15-#16
death
16-#13
autopsy
lung
adrenal gland
lipoma
16-#14
autopsy
lung
adrenal gland
tongue
sublingual gland
ovary
trachea
16-#15
stomach
16-#16
autopsy
lung
adrenal gland
pancreas
dorsal tumor
dorsal tumor
gastrointestine
High
Low

Fig. S4A-2.

1 x 10^6 Luc-B6miPSC   B6 mice tail

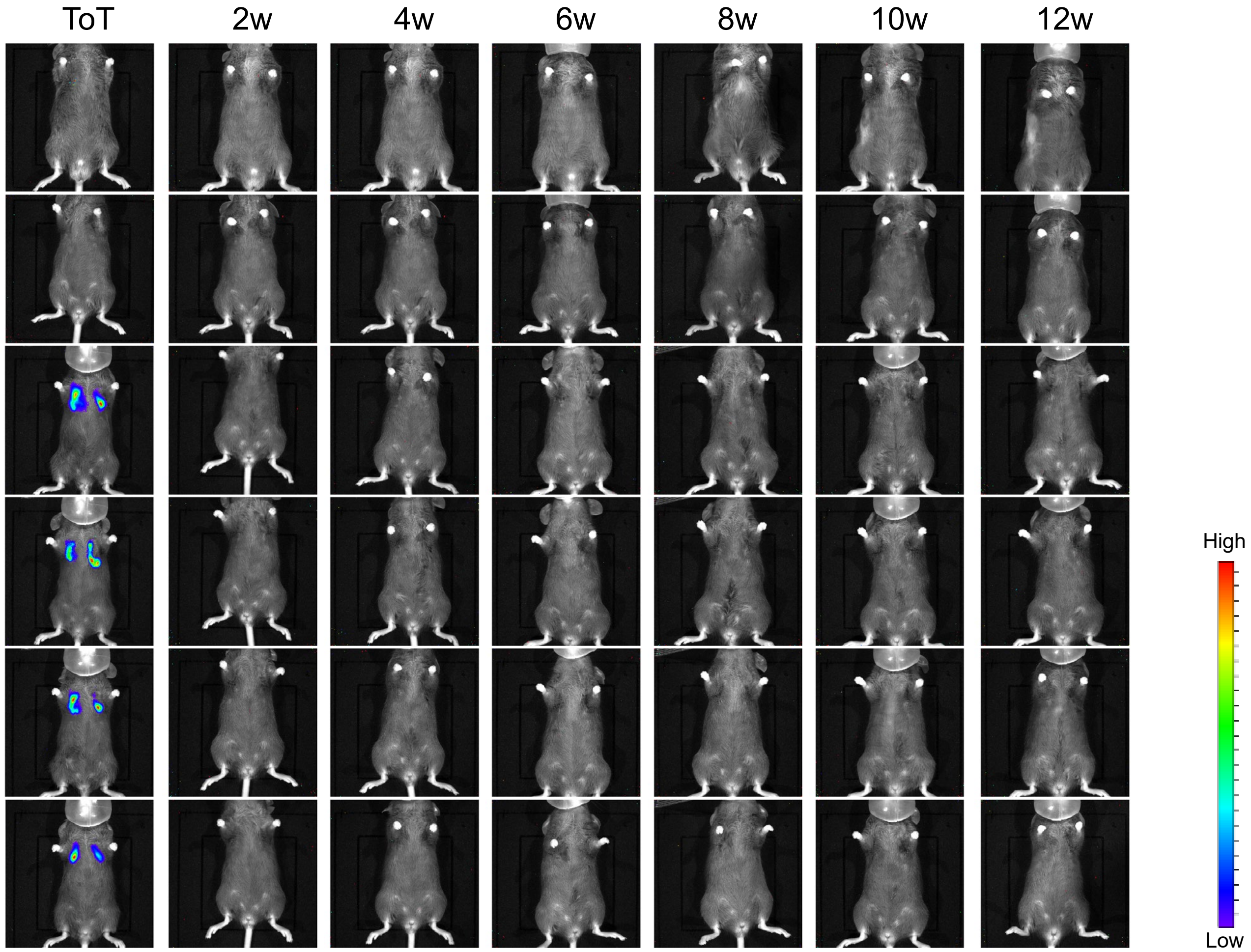

Fig. S4B-1.

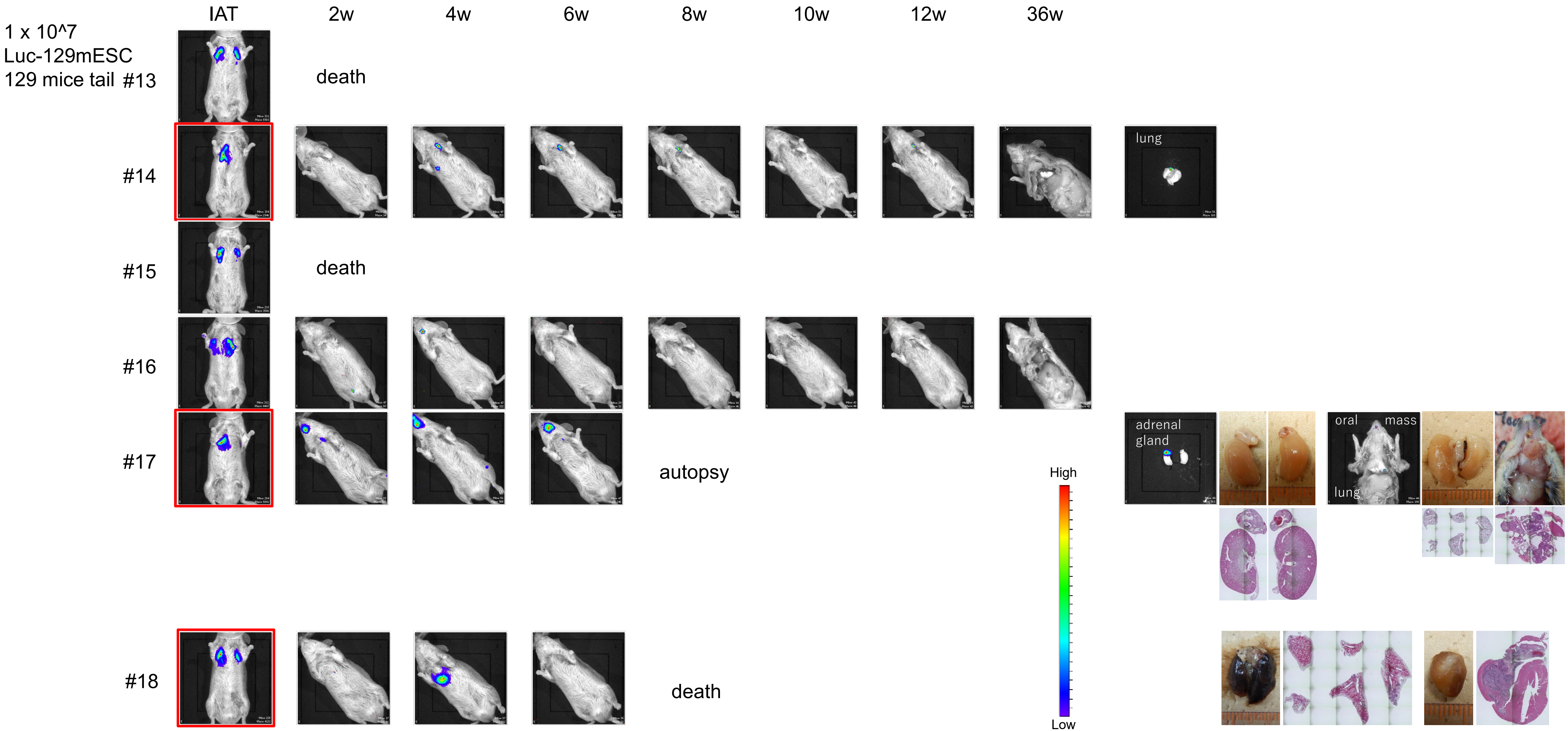

IAT
2w
4w
6w
8w
10w
12w
36w
1 x 10^7
Luc-129mESC
129 mice tail #13
death
#14
lung
#15
death
#16
#17
autopsy
adrenal
gland
oral
mass
lung
High
Low
#18
death

Fig. S4B-2.

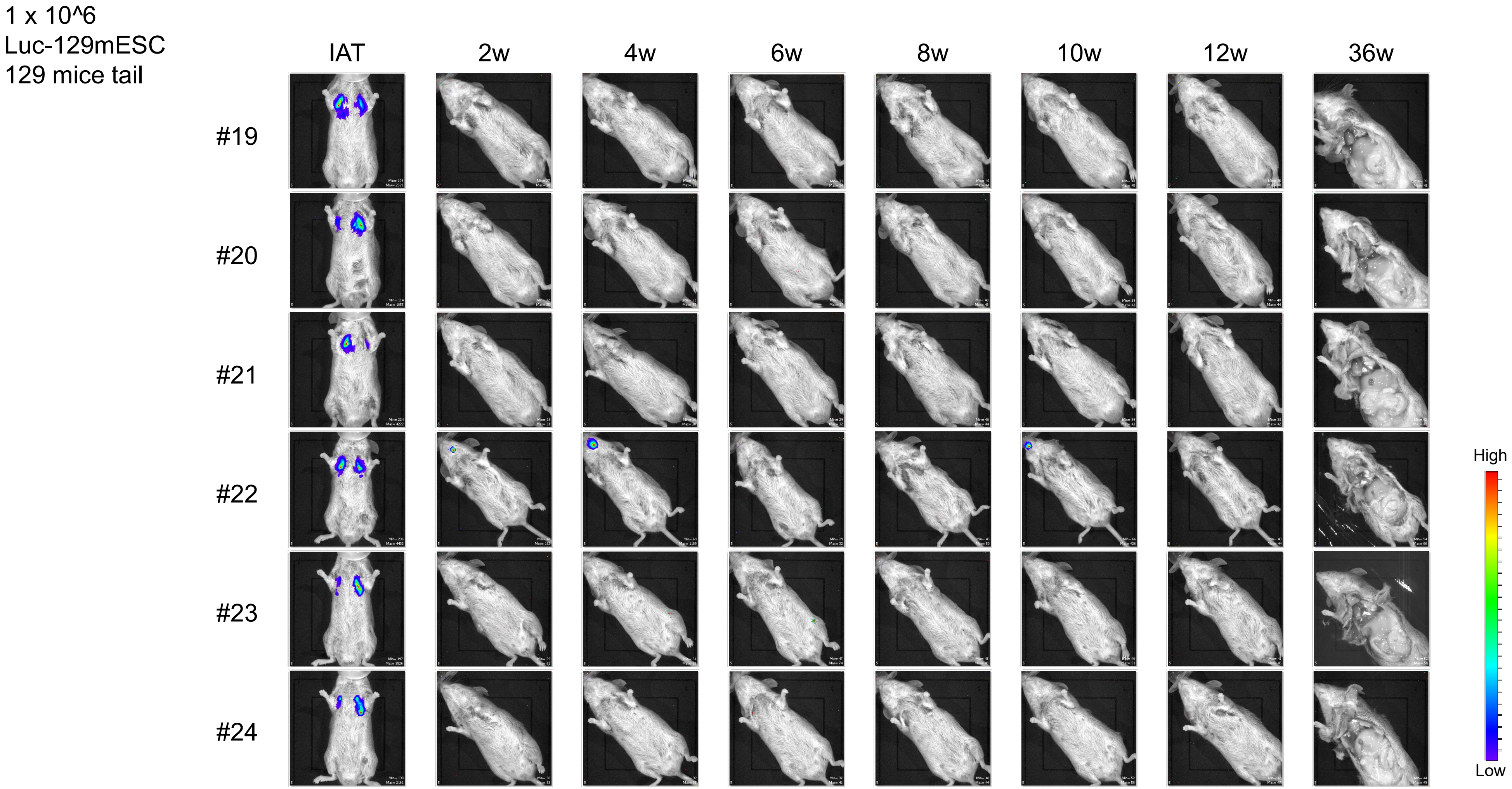
1 x 10^6
Luc-129mESC
129 mice tail
IAT
2w
4w
6w
8w
10w
12w
36w
#19
#20
#21
#22
#23
#24
High
Low

Fig. S5.

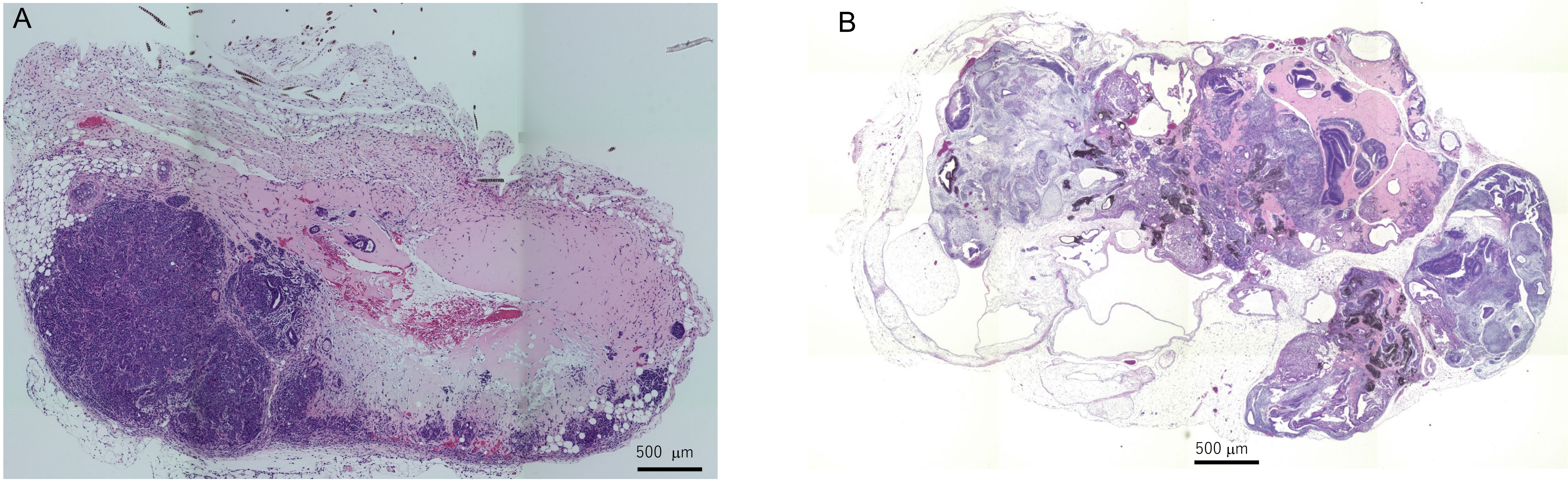

Fig. S5

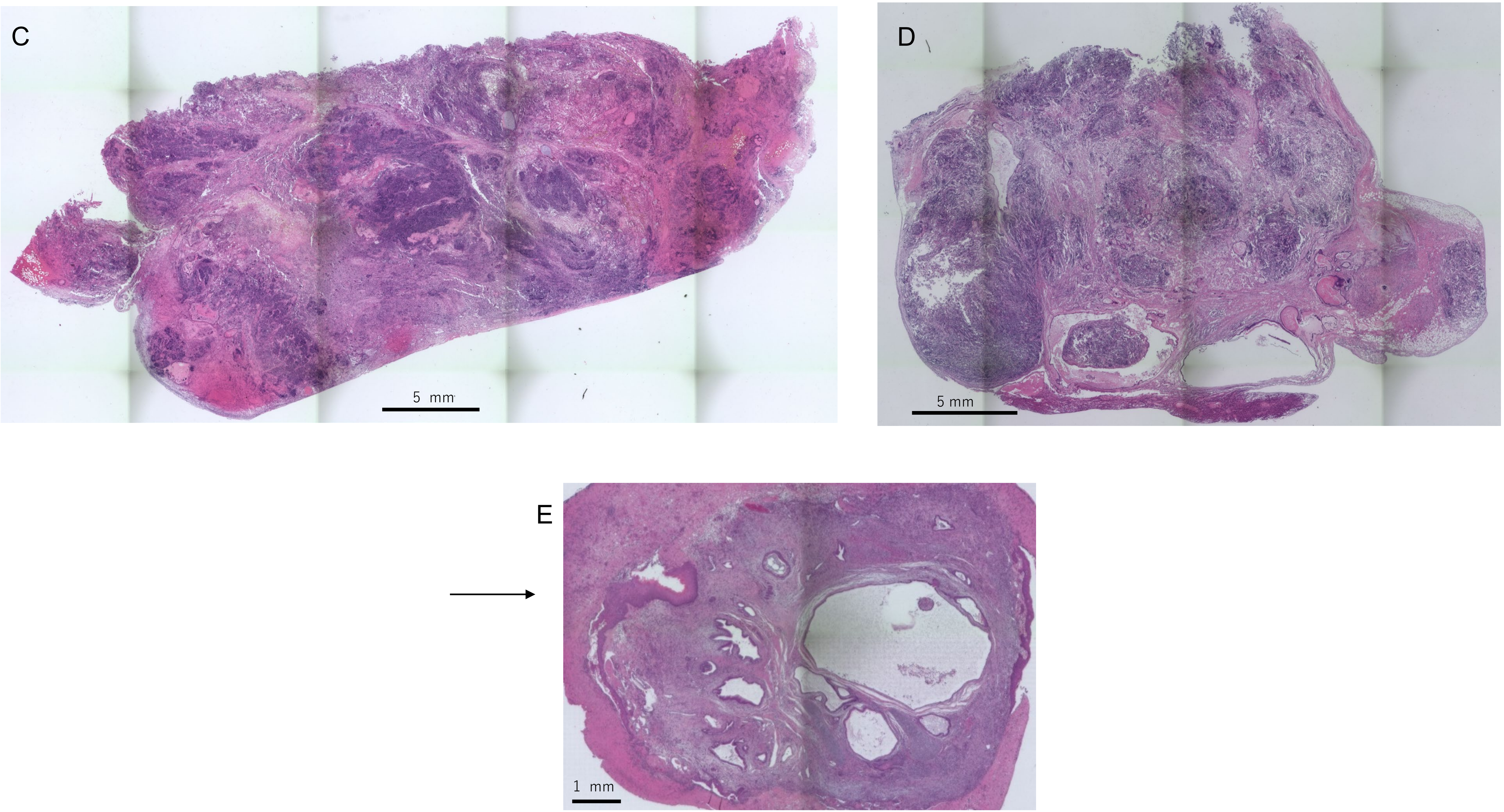

Fig. S5

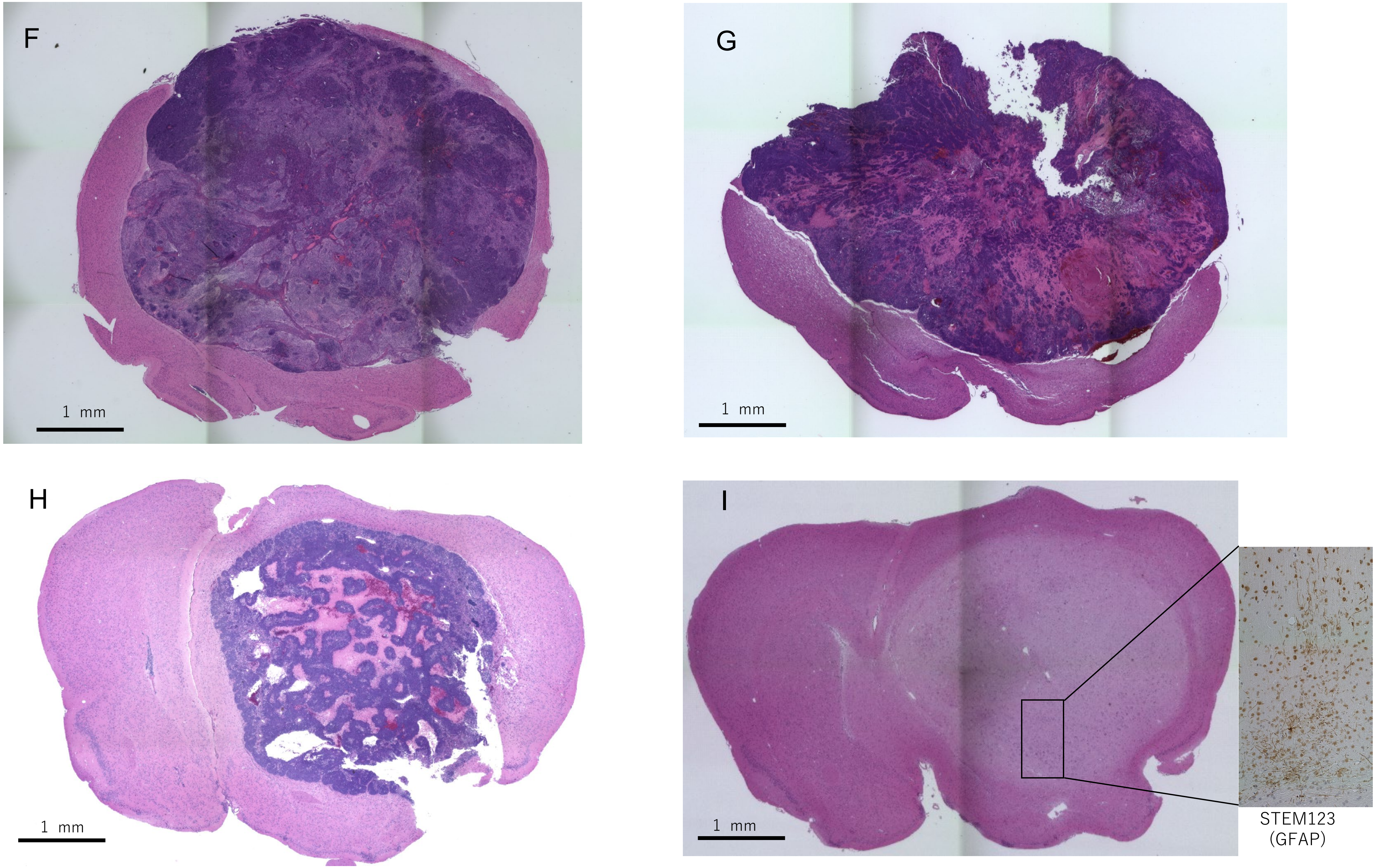

Supplement: szae019_suppl_Supplementary_Material [file szae019_suppl_supplementary_material.zip › Suppl_Figs/Extrapolation of Tumorigenicity test_Supplementary data_R1.pdf]
