# Supplementary material for: Study on the Extrapolability of Current Tumorgenicity Test With Mice by Comparing the Syngeneic or Allogeneic Mouse Transplantation Model
Source: Stem Cells Transl Med. 2024 Jun 10;13(6):572–81. doi: 10.1093/stcltm/szae019 (PMC11165165; doi:10.1093/stcltm/szae019)
Supplement: szae019_suppl_Supplementary_Material [file szae019_suppl_supplementary_material.zip › Suppl_Figs/Suppl_Figure_captions.docx]

Fig. S1. Biweekly IVIS images of B6 mice subcutaneously transplanted with 1×10^2 syngeneic Luc-miPSC-B6 cells. Mice developed teratomas are marked with red rectangles. Observation period was 2-12 weeks. Histology of teratoma generated in subcutaneous and other organs as metastasis in B6 mice are shown respectively.

Fig. S2A-1. 60 min and biweekly IVIS images of liver transplanted with 1×10^5 syngeneic Luc-miPSC-B6 cells. Mice developed teratomas are marked with red rectangles. Observation period was 2-12 weeks. Histology of teratoma generated in different organs as metastasis in B6 mice are shown respectively.

Fig. S2A-2. 60 min and biweekly IVIS images of liver transplanted with 0.5×10^5 syngeneic Luc-miPSC-B6 cells. Observation period was 2-12 weeks. No cell mass with signal was found in this experiment.

Fig. S2B-1. 60 min and biweekly IVIS images of liver transplanted with 1 × 10^5 syngeneic Luc-mESC-129 cells. Mice developed teratomas are marked with red rectangles. Observation period was 2-12 weeks. Histology of teratomas generated in different organs as metastasis in 129 mice are shown respectively.

Fig. S2B-2. 60 min and biweekly IVIS images of liver transplanted with 1×10^4 syngeneic Luc-mESC-129 cells. Observeation period was 2-12 weeks. No cell mass with signal was found in this experiment.

Fig. S2B-3. 60 min and biweekly IVIS images of liver transplanted with 1×10^3 syngeneic Luc-mESC-129 cells. Mouse developed teratoma is marked with red rectangle. Monitoring period was 2-12 weeks. Teratoma found in liver was shown.

Fig. S2C-1. 60 min and biweekly IVIS images of liver transplanted with 1×10^4 Luc-hiPSC-PFX#9 cells. Mouse developed teratoma is marked with red rectangle. Observation period was 2-12 weeks. Teratoma generated in liver and peritoneum are shown respectively.

Fig. S2C-2. 60 min and biweekly IVIS images of liver transplanted with 1×10^3 Luc- hiPSC-PFX#9 cells. No cell mass with signal was found in this experiment.

Fig. S3A-1. IVIS images of brain transplanted with 1×10^3 syngeneic Luc-miPSC-B6 cells. Observation period was 1-12 weeks. Mice developed teratomas are marked with red rectangles. Histology of teratoma generated in different organs as metastasis are shown respectively. Histology of teratomas generated in brain and signal from other organs as metastasis are shown.

Fig. S3A-2. IVIS images of brain transplanted with 1×10^2 syngeneic Luc-miPSC-B6 cells. Mice developed teratomas are marked with red rectangles. Observation period was 1-12 weeks. Histology of teratomas generated in brain and signal from other organs as metastasis are shown.

Fig. S3B-1. Biweekly IVIS images of brain transplanted with 1×10^5 syngeneic Luc-mESC-129 cells. Mice developed teratomas are marked with red rectangles. Observation period was 2-38 weeks. Histology of teratomas generated in brain and signal from other organs as metastasis are shown.

Fig. S3B-2. Biweekly IVIS images of brain transplanted with 1×10^4 syngeneic Luc- mESC-129 cells. Mice developed teratomas are marked with red rectangles. Observation period was 2-38 weeks. Histology of teratomas generated in brain and signal from other organs as metastasis are shown.

Fig. S3C-1. Biweekly IVIS images of brain transplanted with 1×10^4 Luc-mESC-129 cells transplanted in B6 mice (allogeneic transplantation). Mice developed teratomas are marked with red rectangles. Observation period was 2-14 weeks. Teratomas generated in brain and other organs are shown respectively.

Fig. S3C-2. Biweekly IVIS images of brain transplanted with 1×10^3 Luc-mESC-129 cells transplanted in B6 mice (allogeneic transplantation). Mouse with signal at 14 weeks is marked with a red rectangle with enhanced photo. Observation period was 2-14 weeks.

Fig. S3C-3. Biweekly IVIS images of brain of B6 mice transplanted with 1×10^2 Luc- mESC-129 cells (allogeneic transplantation). Mouse with signal at 14 weeks is marked with a red rectangle with enhanced photo. Observation period was 2-14 weeks.

Fig. S3D-1. IVIS images of 1 × 10^4 Luc-labelled hiPSC-PFX#9 cells transplanted into NOG mice (xenotransplantation). Mice developed teratomas are marked with red rectangles. Monitoring period was 2-37 weeks. Histology of teratomas generated in brain are shown.

Fig. S4A-1. Biweekly IVIS images of infused 1×10^7 syngeneic Luc-miPSC-B6 cells. Monitoring period was 2-12 weeks. Mice with signal are marked with red rectangles. Organs with signal were shown.

Fig. S4A-2. Biweekly IVIS images of infused 1 × 10^6 syngeneic Luc-miPSC-B6 cells. Monitoring period was 2-12 weeks. No mouse with signal was observed at 12 weeks.

Fig. S4B-1. Biweekly IVIS images of infused 1 × 10^7 syngeneic Luc-miPSC-B6 cells. Monitoring period was 2-36 weeks. Mice with signal are marked with red rectangles .organs with signal and histology were shown.

Fig. S4B-2. Biweekly IVIS images of infused 1 × 10^6 syngeneic Luc- miPSC-B6 cells. Monitoring period was 2-36 weeks. No mouse with signal was observed at 6-36 weeks.

Fig. S5. Teratoma formation.

Photos of representative teratomas generated from A: Luc-B6miPSC transplanted subcutaneously into B6 mice, B: Luc-hiPSC (PFX#9) transplanted subcutaneously into NOG mice, C: Luc-129mESC transplanted into the liver of 129 mice, D: Luc-B6miPSC transplanted into the liver of B6 mice, E: Luc-hiPSC (PFX#9) transplanted into the liver of NOG mice, F: Luc-B6miPSC transplanted into the striatum of B6 mice, G: Luc-129mESC transplanted into the striatum of B6 mice, H: Luc-129mESC transplanted into the striatum of 129 mice, I: Luc-hiPSC (PFX#9) transplanted into the striatum of NOG mice. SectionsHE and were stained with HE. Sequential slice section was stained with STEM 123 (anti-human GFAP antibody) for the slice section of I.
